# The Influence of Glycosylation of Natural and Synthetic Prenylated Flavonoids on Binding to Human Serum Albumin and Inhibition of Cyclooxygenases COX-1 and COX-2

**DOI:** 10.3390/molecules22071230

**Published:** 2017-07-21

**Authors:** Tomasz Tronina, Paulina Strugała, Jarosław Popłoński, Aleksandra Włoch, Sandra Sordon, Agnieszka Bartmańska, Ewa Huszcza

**Affiliations:** 1 Department of Chemistry, Wrocław University of Environmental and Life Sciences, Norwida 25, 50-375 Wrocław, Poland; jaroslaw.poplonski@upwr.edu.pl (J.P.); sandra.sordon@upwr.edu.pl (S.S.); agnieszka.bartmanska@upwr.edu.pl (A.B.); ewa.huszcza@upwr.edu.pl (E.H.); 2 Department of Physics and Biophysics, Wrocław University of Environmental and Life Sciences, Norwida 25, 50-375 Wrocław, Poland; paulina.strugala@upwr.edu.pl (P.S.); aleksandra.wloch@upwr.edu.pl (A.W.)

**Keywords:** prenylated flavonoids, xanthohumol, microbial glycosylation, glycosides, human serum albumin, cyclooxygenases, COX-1, COX-2

## Abstract

The synthesis of different classes of prenylated aglycones (α,β-dihydroxanthohumol (**2**) and (*Z*)-6,4’-dihydroxy-4-methoxy-7-prenylaurone (**3**)) was performed in one step reactions from xanthohumol (**1**)—major prenylated chalcone naturally occurring in hops. Obtained flavonoids (**2**–**3**) and xanthohumol (**1**) were used as substrates for regioselective fungal glycosylation catalyzed by two *Absidia* species and *Beauveria bassiana*. As a result six glycosides (**4**–**9**) were formed, of which four glycosides (**6**–**9**) have not been published so far. The influence of flavonoid skeleton and the presence of glucopyranose and 4-*O*-methylglucopyranose moiety in flavonoid molecule on binding to main protein in plasma, human serum albumin (HSA), and inhibition of cyclooxygenases COX-1 and COX-2 were investigated. Results showed that chalcone (**1**) had the highest binding affinity to HSA (8.624 × 10^4^ M^−1^) of all tested compounds. It has also exhibited the highest inhibition of cyclooxygenases activity, and it was a two-fold stronger inhibitor than α,β-dihydrochalcone (**2**) and aurone (**3**). The presence of sugar moiety in flavonoid molecule caused the loss of HSA binding activity as well as the decrease in inhibition of cyclooxygenases activity.

## 1. Introduction

Xanthohumol (**1**) (3’-[3”,3”-dimethylallyl]-2’,4’,4-trihydroxy-6’-methoxychalcone) is a major prenylflavonoid found in hop (*Humulus lupulus* L.). It exhibits various biological properties, such as anti-inflammatory, antioxidant, antibacterial, antifungal, anti-plasmodial and antiviral ones [1,2,3]. Besides xanthohumol (**1**), there are also few other biologically active prenylated flavonoids in hops, e.g., isoxanthohumol, 8-prenylnaringenin and α,β-dihydroxanthohumol (**2**). α,β-Dihydroxanthohumol (**2**) is a xanthohumol derivative with hydrogenated α,β-olefinic bond. Its anti-proliferative activity against human breast cancer (MCF-3) and human prostate cancer (PC-3) was tested in vitro. The data showed that α,β-dihydroxanthohumol (**2**) is as strong an anti-proliferative agent as xanthohumol (**1**) and cisplatin—the drug commonly used in cancer treatment [4]. Due to strong desired biological activities, xanthohumol (**1**) is a very attractive starting material for structural modification, which would increase the pharmacological effects in body. Hence, we have used xanthohumol (**1**) as a substrate of chemical synthesis to obtain other prenylated flavonoids: naturally occurring in hops α,β-dihydroxanthohumol (**2**), and synthetic aurone derivative of xanthohumol (**1**)—aurone (**3**). Compound **3** also exhibits high anti-proliferative activity against various human cancer cell lines in vitro (data not published). High therapeutic potential of biologically active aglycones such as xanthohumol (**1**) is strongly limited due to low bioavailability caused possibly by poor solubility in water. Generally, bioavailability of all flavonoids is low and can vary essentially among different flavonoid classes, individual compounds in a particular class as well as among different conjugates of the same compound [5]. Biotransformations conducted by microorganisms are a simple and usually efficient method for obtaining phase I [4,6,7,8,9,10,11] and phase II [7,11,12,13,14,15] biologically active metabolites of flavonoids. Conjugation of bioactive compounds with polar molecules such as sugars, amino acids or sulfuric acid in phase II metabolism leading to increased water solubility is a desired reaction. The presence of sugar moiety in flavonoid molecule was proposed to be the crucial determinant of their absorption in humans [16]. However, it strongly depends on class of flavonoids as well as on the type and position of conjugated sugar moiety. The flavonoids that are most well absorbed in humans are isoflavones [17]. A study performed on the bioavailability of isoflavone, genistein (aglycone) and its glycoside genistin, showed that the bioavailability of the aglycone was higher compared to its glycoside form [5,18], whereas study on absorption of the flavanol quercetin showed the opposite trend. 3-*O*-Glucosylation improved the absorption of quercetin in the small intestine (+184%) compared to quercetin itself in a rat model [19]. The type of attached sugar moiety had a great influence on absorption in humans; quercetin glucosides were absorbed 10 times faster than quercetin rutinosides [16]. These results proved predominant role of glucose moiety in determining bioavailability and absorption of quercetin in the human body and indicated that quercetin glucosides are absorbed from small intestine, whereas the rutinoside form are absorbed from the colon after the deglycosylation catalyzed by colonic microflora [20]. Generally (with some exceptions), glucosides are the only glycosides that can be absorbed from the small intestine after hydrolysis to aglycone form [21]. The intestinal bacteria not only catalyze deglycosylation of glycosides different from glucosides but they are also involved in hydrolysis of glucuronides, and therefore may play a very important role in absorption of flavonoids. Studies on isoflavones absorption showed that major circulating and urinary forms were glucuronides [22,23,24]. Conjugation of glucuronic acid to flavonoid molecule took place in the liver and/or the enterocyte. The study on intestinal perfusion indicate that approximately one-third of the glucuronides formed in small intestine in rat model was hydrolyzed by bacterial glucuronidases abundant in the colon and returned to the lumen [24,25,26], except that colonic bacteria are mostly involved in an intensive degradation of the released aglycone to phenolic acids.

Biotransformations are useful tools for obtaining glycosylated flavonoids from their aglycones conducted by plants and fungal cultures. Application of fungi as biocatalysts allows obtaining glycosylated flavonoids in one-step process in amounts sufficient for biological assays in relatively short time and much cheaper compared to reaction catalyzed by pure glucosyltransferases. Our previous studies on biotransformations of natural prenylated flavonoids from hops showed that *Absidia* genus efficiently catalyze conjugation of β-anomer of glucopyranose, while *Beauveria bassiana* strains are able to attached at the same position in the flavonoid molecule 4-*O*-methyl derivative of β-glucopyranose instead of glucose itself [7,11,12,13,27]. Therefore, we have used fungi mentioned above as biocatalysts for obtaining glycosides **4**–**9** of aglycones **1**–**3**.

Most drugs including flavonoids are transferred to the blood after absorption and then transported in the circulatory system as a complex with plasma proteins such as albumin [28,29,30]. Because only free drugs can cross the biological membrane, the bound drug–albumin complex has no pharmacological effect. However, reversible attachment of drug to plasma proteins significantly modulates the pharmacodynamics (biological activity and toxicology) and pharmacokinetics (volume of distribution, clearance and elimination half-life) of biologically active compounds [29]. Hence, studies of interactions between drugs and plasma proteins have an important role in understanding the transport and distribution of drugs in the body, as well as clarifying the mechanism of action, pharmacokinetics and toxicity of drugs [31].

The aim of the study was to synthesize biologically active prenylated flavonoids (**2** and **3**) from xanthohumol (**1**) to obtain their glycosylated derivatives (**4**–**9**) by means of microbial transformations. The structure-activity relationship was investigated. This article describes how the type of prenylated flavonoid skeleton as well as the presence of sugar moiety affects binding to plasma proteins and inhibition of enzymes associated with generation of inflammation.

## 2. Results and Discussion

### 2.1. Chemistry

#### 2.1.1. Synthesis of α,β-Dihydrochalcone (**2**)

**Figure 1:**
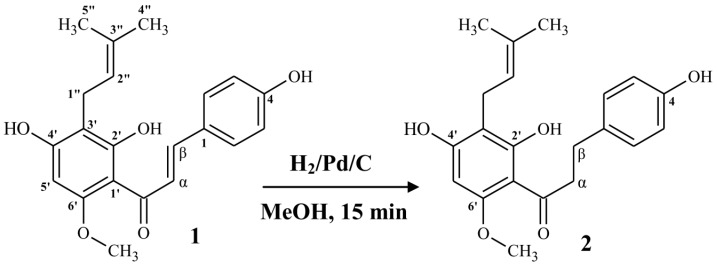
Synthesis of α,β-dihydroxanthohumol (**2**) from xanthohumol (**1**) [34].

α,β-Dihydrochalcone (**2**) occurs naturally in hops as a minor component [32], therefore its isolation is not efficient and economically unjustified. The chemical and biotechnological methods for obtaining this compound are based on regioselective hydrogenation of α,β-olefinic bond in xanthohumol (**1**). As a result of biotransformation of xanthohumol (**1**) by fungi *Fusarium tricinctum* AM16, compound **2** was obtained with 24% yield (14 days incubation) [4], whereas yeasts *Rhodotorula marina* AM77 reduced xanthohumol (**1**) to α,β-dihydrochalcone (**2**) with 18% yield in seven days [33]. The most effective method is chemical, regioselective hydrogenation of xanthohumol (**1**) in the presence of palladium on charcoal catalyst and H_2_ gas as a donor of hydrogen atoms [34]. Due to the highest yield of α,β-dihydrochalcone (**2**), we decided to use the method proposed by Popłoński et al. (Figure 1) [34]. The spectral characteristic of obtained compound (**2**) was in agreement with literature data [4,34].

#### 2.1.2. Synthesis of (*Z*)-6,4’-Dihydroxy-4-methoxy-7-prenylaurone (**3**)

To the best of our knowledge, (*Z*)-6,4’-dihydroxy-4-methoxy-7-prenylaurone (**3**) has been previously reported in the literature only once. It was obtained with poor yield (6.2%) as the major product of oxidation of xanthohumol (**1**) with 3-morpholinosydnonimine (SIN-1) [35]. Using xanthohumol (**1**) as a substrate and the method of cyclization of 2’-hydroxychalcones to *Z*-aurones by Venkateswarlu et al. [36], we produced (*Z*)-6,4’-dihydroxy-4-methoxy-7-prenylaurone (**3**) with significantly higher yield (64.8%). Ten times more efficient method of synthesis of (*Z*)-6,4’-dihydroxy-4-methoxy-7-prenylaurone (**3**) allowed us to subject it to biological tests.

The structure of (*Z*)-6,4’-dihydroxy-4-methoxy-7-prenylaurone (**3**) was confirmed by NMR, mass spectroscopy and spectrophotometry.

**Figure 2:**
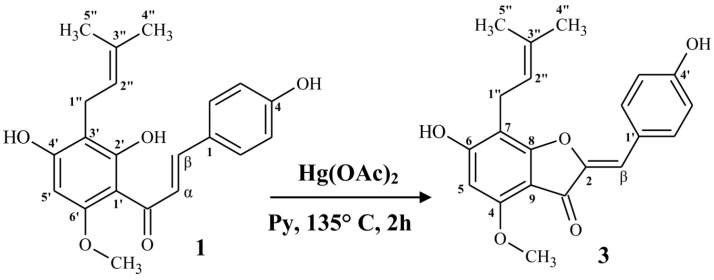
Synthesis of (*Z*)-6,4’-dihydroxy-4-methoxy-7-prenylaurone (**3**) from xanthohumol (**1**).

As a result of chalcone-aurone type cyclization, new five-membered heterocyclic ring was formed, which required renumbering of all carbon atoms in the product, except for the prenyl moiety (Figure 2). Aurones absorb light of longer wavelength than any other closely related flavonoid pigments such as flavanones, flavones or chalcones. The low absorption of the aurone chromophore is attributed to the cross conjugation between coumaranone moiety and ring B [37]. Large bathochromic shift (31.5 nm) of the major band in the UV spectrum of **3** compared to chalcone-xanthohumol (**1**) (Figure S3) unambiguously confirms formation of an aurone. High-resolution electrospray ionization mass spectral data (Figure S4) showed the molecular ion [M − H]^−^ at *m*/*z* 351.1258 which established the molecular formula as C_21_H_20_O_5_ (calcd. for C_21_H_20_O_5_ − H, 351.1238).

In the ^1^H-NMR spectrum of **2**, there are no olefinic proton signals visible, characteristic for a chalcone (H-α, H-β), whereas new singlet at δ 6.58 ppm was observed (Figure S6) and showed interactions with benzilidene carbon signal at δ 109.5 ppm in the ^1^H–^13^C-NMR (HSQC) correlation spectrum (Figure S9). In the ^13^C-NMR spectrum of compound **3** we observed the following signal shifts compared to **1**: C-α from δ_H_ 123.8 ppm to δ_C_ 146.0 ppm (C-2), C-β from δ_H_ 142.6 ppm to δ_C_ 109.5 ppm (C-benzilidene) and the carbonyl carbon signal from δ_C_ 191.7 ppm to δ_C_ 179.0 ppm (Figure S7). The carbonyl carbon signal appears at so low field (δ_C_ 191.7 ppm) in the spectrum of xanthohumol (**1**) because of the intramolecular hydrogen bond that forms between the oxygen atom (C=O) and the hydrogen atom of the hydroxyl group at C2’. Due to direct involvement of C2’-hydroxyl group in the reaction of cyclization of chalcone-aurone type, the hydrogen bond is cleaved and the hydroxyl group is engaged in formation of a new five-membered heterocyclic ring. Therefore, the carbonyl carbon atom signal in the spectrum of product **3** is found at significantly higher field (δ_C_ 179.0 ppm) and the signal of C-2’ hydroxyl proton present in the spectrum of xanthohumol (**1**) at δ_C_ 14.65 ppm does not exist in the spectrum of **3** (Figure S6). The chemical shift value of benzylidene carbon at 109.5 ppm is in agreement with data reported for other (*Z*)-aurones (δ_C_ 108.1–112.8 ppm), while the resonances of (*E*)-aurones are known to range from δ_C_ 119.8 to 122.2 ppm [38].

All the NMR, MS and spectrophotometric data indicated that an aurone-type flavonoid was formed from the chalcone-type flavonoid and the data fully confirmed the structure of (*Z*)-6,4’-dihydroxy-4-methoxy-7-prenylaurone (**3**) as a product of cyclization of xanthohumol (**1**).

### 2.2. Regioselective Microbial Glycosylation of Prenylated Flavonoids

**Figure 3:**
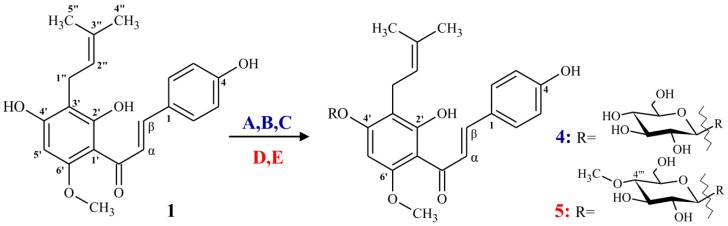

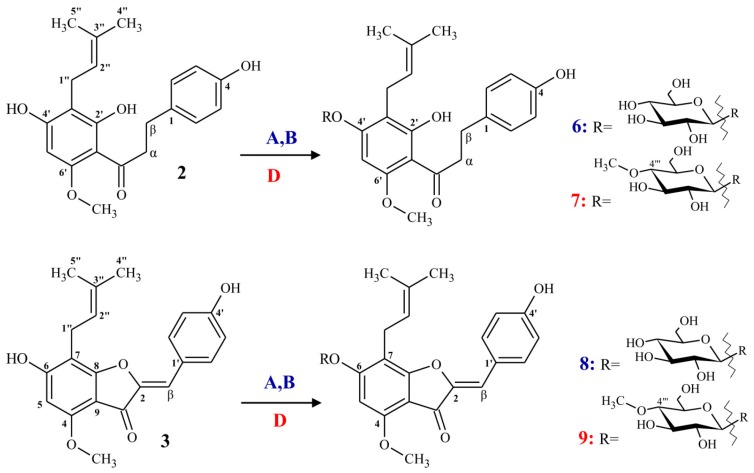
Fungal transformations of prenylated flavonoids 1-3 catalyzed by: (**A**) *Absidia coeruela* AM93; (**B**) *Absidia glauca* AM177; (**C**) *Rhizopus nigricans* UPF701; (**D**) *Beauveria bassiana* AM278; and (**E**) *B. bassiana* AM446.

Our previous studies on microbial metabolism of flavonoids showed that the fungal strains *Absidia glauca* AM177, *A. coerulea* AM93, *Rhizopous nigricans* UPF701, *Beauveria bassiana* AM278 and *B. bassiana* AM446 are able to conjugate sugar moiety to chalcones, flavanones and isoflavanones with high regioselectivity. Therefore we decided to used fungi belonging to *Beauveria* and *Absidia* genera for microbial transformation of naturally occurring, minor prenylated hop flavonoid—α,β-dihydroxanthohumol (**2**) and synthetic aurone derivative (**3**) of xanthohumol (**1**). As results of biotransformations, we have obtained glycosides of prenylated flavonoids (**6**–**9**). As expected, strains belonging to *Absidia* genus were able to attach glucopyranose, while *Bauveria basisana* AM278 catalyzed conjugation of 4-*O*-methyl derivative of glucopyranose (Figure 3). To our best knowledge, the obtained flavonoid glycosides (**6**–**9**) are novel and have not been published so far.

#### 2.2.1. Biotransformations Catalyzed by *Absidia coeruela* AM93 and *A. glauca* AM177

Strains *Absidia coeruela* AM93 and *A. glauca* AM177 were able to catalyze conjugation of β-anomer of glucopyranose molecule to both tested substrates (**2** and **3**). Our previous studies revealed that the preferable position of attaching sugar moiety by fungi belonging to *Abisidia* genus is hydroxyl group at C7 in A-ring of flavonoid (due to different numbering of different flavonoid skeletons the same position is designated as C4’ in case of chalcones and α,β-dihydrochalcones, and C6 in aurones) [7,12,13,15,27]. As a result of 10-day incubation of α,β-dihydrochalcone **2** in A. *coeruela* AM93 culture, glycoside **6** was obtained with high yield (70%), while 9-day biotransformation of aurone **3** by *A. glauca* AM177 resulted in formation of its glycosylated derivative **8** with yield close to 48%. The structures of both glycosides were confirmed by NMR and MS methods. Several remarkable differences were observed in the ^1^H-NMR and ^13^C-NMR spectra of both glycosides **6** and **8** compared to their aglycones **2** and **3**. The presence of new signals (mostly overlapping) at ^1^H-NMR in the region ranging δ_H_ 3.0–5.1 ppm and six new signals in range δ_C_ 60–103 ppm of ^13^C-NMR spectra of metabolites **6** and **8** (Figures S12 and S22), confirms the presence of hexose moiety. Chemical shift values of signals, which appeared in ^13^C-NMR spectra of compound **6** and **8** clearly indicates that the sugar attached to both substrates (**2** and **3**) was glucopyranose. The presence of: (a) doublets at chemical shift δ_H_ 5.09 ppm and δ_H_ 5.08 in ^1^H-NMR of **6** and **8** spectra, respectively; and (b) signals at δ_C_ 101.5 ppm and δ_C_ 102.1 in ^13^C-NMR of **6** and **8** spectra, respectively, indicates without any doubts, that conjugated sugar had β-configuration (Figures S12 and S22). The correlations between protons and carbon atoms presented on ^1^H–^13^C-NMR (HSQC) spectra allowed to assign accurate position of each proton signal despite many of them being overlapped on each other (Figures S14 and S24). The shift in position of signals H-5’ (α,β-dihydrochalcone **6**) and H-5 (aurone **8**) in ^1^H-NMR spectra towards the lower magnetic field, H-5’ δ_H_ 5.98 **→** 6.46 ppm and H-5 δ_H_ 6.23 **→** 6.60 ppm clearly indicates that sugar moiety was attached to hydroxyl group at C4’ (α,β-dihydrochalcone **6**) and C-6 (aurone **8**). The rest of the ^1^H and ^13^C-NMR signals in spectra of metabolites **6** and **8** were very similar to those of substrates **2** and **3** suggesting that the introduction of sugar moiety to ring A was the only reaction catalyzed by two tested *Absidia* species.

#### 2.2.2. Biotransformations Catalyzed by *Beauveria bassiana* AM278

Fungus *Beauveria bassiana* is frequently used as a whole cell catalyst. Over 300 different compounds have been successfully modified by means of biotransformation by *B. bassiana* so far. It is able to catalyze wide range of reactions including hydroxylation, oxidation, reduction and hydrolysis [39]. It is also known that *B. bassiana* catalyzes reaction of glycosylation of numerous compounds by attaching 4-*O*-methylated derivative of glucopyranose to the substrate molecule [40,41,42,43,44]. Our previous studies showed that conjugation of 4-*O*-methyl-glucopyranose with prenylated and none-prenylated flavonoids was the major reaction in case of biotransformation conducted by *B. bassiana* [7,12,13,15,27]*.* 4-*O*-methylglycosylation reaction has been also observed in biotransformations of α,β-dihyroxanthohumol **2** and aurone **3**, as a result two glycosides **7** and **9** were obtained. The structures of products were determined by NMR and MS methods.

HR ESI-MS spectrum of metabolite **7** showed the [M − H]^−^ peak at *m/z* 531.2236, which was consistent with the molecular formula of C_28_H_36_O_10_ (calculated for C_28_H_36_O_10_ − H ([M − H]^−^ 531.2236)), whereas spectrum of metabolite **9** showed the [M + H]^+^ peak *m/z* 529.2077 which established a molecular formula of C_28_H_32_O_10_ (calculated for C_28_H_32_O_10_ + H ([M + H]^+^ 529.2068). It proves that compared with aglycones **2** and **3**, metabolites **7** and **9** contain seven additional carbon atoms.

The presence of new overlapping signals at ^1^H-NMR in the region ranging δ_H_ 3.0–5.1 ppm and seven new signals in range δ_C_ 59–102 ppm of ^13^C-NMR spectra of metabolites **7** and **9** (Figures S17 and S27) suggests that sugar moiety was attached to the substrate molecules. Values of chemical shifts of five from seven newly appeared signals in ^13^C-NMR spectra of metabolites **7** and **9** are characteristic and typical for β-anomer of glucopyranose moiety with an exception of position of signal C4 (C4’’’), which normally is present at range δ_C_ 70.0–71.5 ppm in glucopyranose but in spectra of metabolites **7** and **9** was shifted into the lower field δ_C_ 71.5 **→** 80.6 ppm (metabolite **7**) and δ_C_ 71.5 **→** 76.1 ppm (metabolite **9**). These data indicate that not a glucose itself but its C4 (C4’’’)-substituted derivative was involved in glycosylation reaction. Compared with glycosides **6** and **8**, metabolites obtained in *B. bassiana* culture contain one additional carbon atom, which is present in ^13^C-NMR spectra of metabolites **7** and **9** at δ_C_ 60.6 ppm and at δ_C_ 59.7 ppm, respectively. This carbon atom showed correlation in HSQC spectra with three protons singlet at δ_H_ 3.55 (correlation with δ_C_ 60.6 ppm—compound **7**) and δ_H_ 3.46 (correlation with δ_C_ 59.7 ppm—compound **9**), which confirms the presence of methoxyl group. In conclusion, the values of chemical shifts of seven additional carbon atoms which appeared in ^13^C-NMR spectra of metabolites **7** and **9** but not in aglycones **2** and **3**, are characteristic for 4-*O*-methyl-glucopyranose moiety, the sugar which is very often involved in glycosylation reactions catalyzed by *B. bassiana.* The shift in position of signals H-5’ (α,β-dihydrochalcone **7**) and H-5 (aurone **9**) in ^1^H-NMR spectra towards the lower magnetic field, H-5’ δ_H_ 5.98 → 6.34 ppm and H-5 δ_H_ 6.23 → 6.55 ppm clearly indicates that sugar moiety was attached to hydroxyl group at C4’ (α,β-dihydrochalcone **7**) and C6 (aurone **9**). The rest of the signals in ^1^H and ^13^C-NMR spectra of metabolites **7** and **9** were not shifted and had chemical shifts very similar to those of substrates **2** and **3** suggesting the 4-*O*-methylglycosylation was the only reaction catalyzed by *B. bassiana* AM278.

Metabolites (**6**–**9**), xanthohumol glycosides (**4** and **5**) obtained by us previously [12], and substrates used in biotransformations (**1**–**3**) were tested in human serum albumin fluorescence quenching assay and inhibition of cyclooxygenase activity of COX-1 and COX-2 assay.

### 2.3. Binding to Human Serum Albumin

#### 2.3.1. Fluorescence Quenching Mechanism of HSA

**Figure 4:**
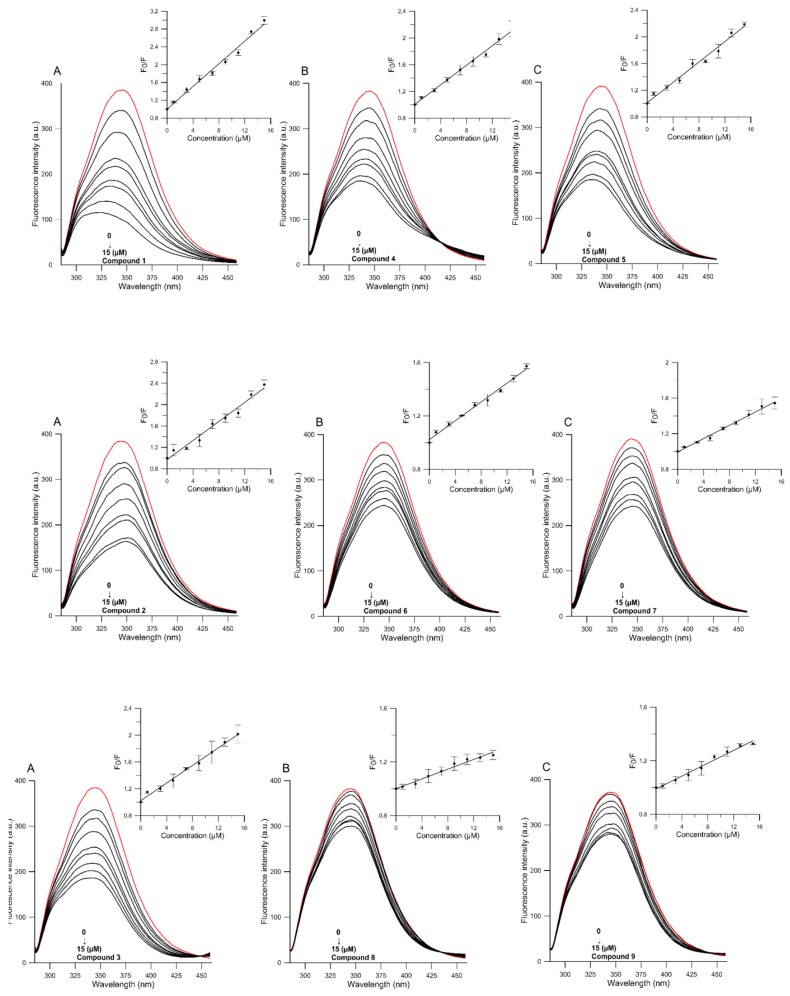
Emission spectra of HSA in the presence of various concentrations of tested compounds and Stern-Volmer plots of *F*_0_*/F* against the concentration of tested compounds (HSA = 1.5 × 10^−5^ M, λ_ex_ = 280 nm, T = 310 K). Control is marked red and consecutive spectra of the studied compounds (marked grey) are in the following concentrations 1, 3, 5…, 15 µM. Top graphs present xanthohumol (**1**) and its glycosides (**4**–**5**); middle graphs present α,β-xanthohumol (**2**) and its glycosides (**6**–**7**); and bottom graphs present aurone **3** and its glycosides (**8**–**9**) ((**A**) aglycones (**1**–**3**); (**B**) glycosides (**4**, **6**, and **8**); and (**C**) 4-*O*-methylglycosides (**5**, **7**, and **9**)).

The fluorescence spectra of HSA in the absence and presence of tested compounds were recorded. It is apparent in Figure 4 that the fluorescence intensity of HSA decreased with increasing compound concentration, which suggests that compounds quench the intrinsic fluorescence of the HSA.

Modifications in fluorescence emission spectra of HSA are indicative of the changes around the microenvironment of tryptophan residue (Trp). Blue shift of the emission peak is related to the exposition of Trp to a more hydrophobic environment while a red shift indicates that the microenvironment of Trp residue became more hydrophilic [45]. Xanthohumol (**1**) induced considerable blue shift of the HSA emission maximum relative to compound concentration. The blue shift of the maximum emission wavelength suggests a reduction in the polarity of the microenvironment around fluorophore [46].

The following Stern-Volmer equation [47] was used to analyze the fluorescence quenching mechanism induced by tested compounds at different temperatures (300, 305, 310 and 315 K).

(1) $$\frac{F_{0}}{F}=1+K_{q}\tau_{0}\left[Q\right]=1+K_{SV}\left[Q\right]$$

where *F*_0_ and *F* are the fluorescence intensities of HSA in the absence and presence of tested compounds respectively, *K_q_* is a bimolecular quenching constant, τ_0_ is the life time of the fluorophore in the absence of quencher (the fluorescence life time of a biopolymer is about 5 × 10^−9^ s), [*Q*] is concentration of the quencher studied compounds, and *K_SV_* is the Stern-Volmer quenching constant (*K_SV_ = K_q_·*τ_0_) [48]. Based on Equation (1), the Stern-Volmer quenching constant (*K_SV_*) for the ligand–protein complex was determined using the linear regressions of the plots of *F*_0_*/F* versus [*Q*]*.* The plots were linear in the 1–15 μM range of concentration for all the compounds (Figure 4).

Quenching can occur by different mechanisms which are usually classified as dynamic quenching and static quenching. Dynamic and static quenching can be distinguished by their differing dependence on temperature and viscosity, or preferably by lifetime measurements [47]. Fluorescence quenching can be static, resulting from the formation of a ground-state complex between the fluorophore and the quencher or dynamic, resulting from collisional encounters between the fluorophore and the quencher. Higher temperatures will typically result in the dissociation of weakly bound complexes, and hence smaller amounts of static quenching.

The results showed (Table 1) that the values of Stern-Volmer quenching constants K_SV_ decreased with increasing temperature which indicated that the probable quenching mechanism of drug–HSA interaction was initiated by complex formation.

Second of the criteria for determination of a static quenching mechanism is that the value of *K_q_* is higher than the value of a diffusion limited rate constant of a biomolecule (1.0 × 10^10^ M^−1^ s^−1^). The values of *K_q_* (Table 1) for binding of all tested systems were higher than 1.0 × 10^10^ M^−1^ s^−1^ indicating that the quenching was mainly static, due to complex formation between HSA and studied compounds.

#### 2.3.2. Binding Constant *K_b_* and Number of Binding Sites

In general, the binding constant *K_b_* reflects the power of ligand–protein association and thus can be used for comparison of the binding affinities of structurally-related ligands to a protein determined by the alterations in its secondary structure [48].

The apparent binding constant (*K_b_*) and number of binding sites (*n*) can be calculated using the following equation:

(2) $$log\left(\frac{F_{0}-F}{F}\right)=logK_{b}+nlog\left[Q\right]$$

The values of *n* and *K_b_* were obtained by plotting log [(*F*_0_-*F)*/*F*] versus log [*Q*]. The results for tested compounds as *Q* (as the quencher) at four different temperatures (300, 305, 310 and 315 K) are given in Table 1. The results showed that the values of *K_b_* decreased with increasing temperature. This indicates that the increase of temperature decreases the stability of the compound—HSA complex.

Drugs bind to high affinity sites with typical association constants in the range of 10^4^–10^6^ M^−1^ [29]. The strongest binding affinity at the lowest tested temperature (300 K) was observed for xanthohumol (**1**) (8.624 × 10^4^ M^−1^) (Table 1). The hydrogenation of α,β-olefinic bond in xanthohumol (**1**) to α,β-dihydroxanthohumol (**2**) (3.635 × 10^4^ M^−1^) as well as cyclization chalcone–aurone type (xanthohumol (**1**) to aurone (**3**) (1.425 × 10^4^ M^−1^)) resulted in the significant decrease of binding affinity. It suggests that the skeleton of prenylated aglycones has a great importance in binding to human plasma proteins.

The importance of the presence of the sugar moiety in the A-ring of flavonoids was evident. Quenching constants for all tested glycosides (**4**–**9**) were significantly lower compared to their aglycones (**1**–**3**) (Table 1). The lower values of binding constant of tested glycosides (**4**–**9**) can be explained by structure to binding affinity relationship which showed that the presence of large sugar moiety in the flavonoid molecule generated steric hindrance in the binding pocket. The sugar moiety also increased polarity of the molecule, lessening the ability of the flavonoid glycosides to penetrate into the Trp-rich hydrophobic interior regions of HSA, therefore significantly weakening the binding affinity. The same trend was also observed for different flavonoids, where glycosylation reduced the affinity of flavonoids for human serum albumin by 1–2 orders of magnitude [30].

The influence of additional 4-*O*-methyl group in glucopyranose moiety on binding affinity is not clear. Comparing pairs of glycosides and 4-*O*-methylglycosides from the same precursor, the binding affinity is higher in case of glycosylated prenylated chalcone (**4** and **5**), while glycosylated prenylated aurones (**8** and **9**) showed opposite trend. The binding affinity values of prenylated dihydrochalcone conjugated with glucopyranose (**6**) and 4-*O*-methylglucopyranose (**7**) are comparable.

It seems that the steric hindrance, hydrophobicity and spatial arrangement are key factors in the affinity of flavonoids towards plasmatic proteins [48,49,50].

Number of binding sites determined for tested compound—HSA complexes ranged from 0.515 to 0.971 suggesting that one molecule of HSA associated with one molecule of given compounds.

#### 2.3.3. Thermodynamic Parameters Measurements

In the drug-protein binding process, there are four main types of non-covalent interactions: hydrogen bonds, hydrophobic effects, electrostatic interactions and van der Waals forces [51]. The magnitude of the thermodynamic parameters such as the free energy change (*∆G*), enthalpy change (*∆H*), and entropy change (*∆S*) of the reaction are important for the determination of the interaction forces. The thermodynamic parameters can be calculated by the Van’t Hoff’s equation [30]:

(3) $$lnK_{b}=\frac{-\Delta H}{RT}+\frac{\Delta S}{R}$$

The enthalpy change (*∆H*) is calculated from the slope of the van’t Hoff relationship and the free energy change (*∆G*) is then estimated from the following relationship:

(4) $$\Delta G^{0}=\Delta H-T\Delta S=-RTlnK_{b}$$

where *ΔH*, *ΔG* and *ΔS* are enthalpy change, free enthalpy change and entropy change, respectively. *R* is the gas constant 8.314 J mol^−1^ K^−1^.

The values of *∆G*, *∆H* and *∆S* obtained at different temperatures are shown in Table 1. The interaction between HSA and tested flavonoids had a negative value of *∆G*, *∆H* and *∆S*. The negative values of free energy (*∆G*), as shown in Table 1, support the assertion that the binding processes are all spontaneous.

The model of interaction between the compound and HSA can be determined as follows:

(a) *∆H* > 0 and *∆S* > 0, hydrophobic forces;
(b) *∆H* < 0 and *∆S* < 0, van der Waals interactions and hydrogen bonds; and
(c) *∆H* < 0 and *∆S* > 0, electrostatic interactions [52,53].

Therefore, the negative *∆H* and *∆S* values suggest that all compounds bind to HSA mainly through hydrogen bonds and van der Waals forces. The negative value of *∆G* indicates that the reaction is spontaneous.

### 2.4. Inhibition of Cyclooxygenases (COX-1 and COX-2) Activity

Constitutive cyclooxygenase 1 (COX-1) is responsible for prostaglandins production in digestive tract that are involved in mucosal protection and other physiological activities. Inducible cyclooxygenase 2 (COX-2) is involved in production of prostaglandins that mediate inflammation including fever and pain [54]. Xanthohumol (**1**) was previously reported to have high anti-inflammatory activity [55], therefore it was interesting to determine how the skeleton of prenylated flavonoid (compounds **1**–**3**) and the presence of glucopyranose (compounds **4**, **6**, and **8**) or 4-*O*-methylglucopyranose moiety (compounds **5**, **7**, and **9**) affect inhibition of enzymes involved in inflammatory reactions. Hence, aglycones xanthohumol (**1**) α,β-dihydroxanthohumol (**2**) and aurone **3** as well as their glycosides (**4**–**9**) were evaluated for their inhibition activity towards the two cyclooxygenases: the constitutive form COX-1 and the inducible COX-2. The results were calculated as the IC_50_ (μM), i.e., the concentration of tested agent that inhibits activity of enzyme by half (Table 2).

Xanthohumol (**1**) appeared as the strongest inhibitor of both tested cyclooxygenases. Inhibition activity of xanthohumol (**1**) was comparable for COX-1 and COX-2. The same trend was observed for others tested flavonoids, besides glycosides **4** and **5** which exhibited higher inhibitory activity on COX-2.

The type of flavonoid skeleton and the presence of sugar moiety in flavonoid molecule are the major structural determinants of inhibitory activity. The reduction of α,β double bond as well as cyclization chalcone-aurone type is not desired. Both xanthohumol derivatives α,β-dihydrochalcone **2** and aurone **3** exhibited two-fold lower inhibition activity compared to chalcone **1**. The presence of large polar sugar moiety conjugated with prenylated flavonoid caused the decrease of their activity up to 3–4 fold in the case of aglycones **2**, **3** and close to six fold in the case of glycosides **4**, **5** compared to xanthohumol (**1**).

Despite wide ranges of biological activities of chalcones, α,β-dihydrochalcones and aurones the studies on bioavailability and absorption of aglycones and their glucosides are very limited due to low abundance in nature. Absorption of α,β-dihydrochalcones-phloretin and its 2’-*O*-β-d-glucoside (phloridzin) using an intestinal perfusion model in situ was investigated [56]. The data showed that both α,β-dihydrochalcones were absorbed in the small intestine of rats. The hydrolysis of sugar moiety was the crucial step in the intestinal metabolism. In the following study, Crespy et al. [57] compared the changes in plasma and urine concentrations of both flavonoids in rats. The bioavailability of phloretin and its glucoside was found to be similar. However, the plasma kinetics of phloretin and phloridzin were different [20,57].

The studies on bioavailability of different classes of flavonoid glycosides showed that glucosides were mostly absorbed in small intestine, whereas glycosides such as rutinosides, maltosides, etc., despite their higher water solubility, were barely absorbed. That indicates that absorption of such glycosides took place in colon after hydrolysis to aglycones by colonic bacteria. To our best knowledge the studies on bioavailability of glycosylated prenylflavonoids has not been published so far. Therefore, it is very difficult to assume how the presence of prenyl group in glycosylated flavonoids affects absorption and bioavailability in body. The study of bioavailability and absorption of prenylated glycosylated flavonoids compared to their aglycones in vivo is the goal of our next research.

## 3. Material and Methods

### 3.1. General Experimental

Reagents and solvents (analytical or HPLC grade) were purchased from Sigma-Aldrich (St. Louis, MO, USA), Merck (Darmstadt, Germany) or POCH (Gliwice, Poland). TLC was carried out on Merck silica gel 60, F254 (0.2 mm thick) plates with solvent mixtures specified in each experiment. After drying, spots were visualized under short- and long-wavelength UV light, then plates were sprayed with methanol–sulfuric acid (1:1, *v*/*v*) solution. Column chromatography was performed using either Sephadex LH-20 (GE Healthcare, Uppsala, Sweden) or silica gel 60 (230−400 mesh ASTM, Fluka, Buchs, Switzerland) with solvent mixtures specified in each experiment. HPLC was performed on a Waters 2695 Alliance instrument (Milford, MA, USA) with a photodiode array detector Waters 2996 (detection from 220 to 450 nm wavelength) using the analytical HPLC column Agilent ZORBAX Eclipse XDB (Agilent, Santa Clara, CA, USA) 5 µm (46 mm × 250 mm) at the flow rate of 1 mL/min. A linear solvent gradient from 40 to 60% aq MeCN containing 1% HCOOH over 40 min was used. UV spectra were recorded on a spectrophotometer Cintra 303, (GBC Scientific Equipment, Braeside, VIC, Australia), in methanol. ^1^H-NMR, ^13^C-NMR, DEPT 135°, ^1^H–^1^H-NMR (COSY), and ^1^H–^13^C-NMR (HSQC and HMBC) were recorded on a DRX Bruker Avance II 600 (600 MHz) or DRX Bruker Avance 300 (300 MHz) instrument (Bruker, Billerica, MA, USA) in DMSO-*d*_6_, acetone-*d*_6_ or CD_3_OD. Positive and negative-ion HRESI-MS spectra were recorded on a Bruker Daltonics microTOF-Q spectrometer (Bruker, Billerica, MA, USA). The direct infusion of ESI-MS parameters: the mass spectrometer was operated in negative or positive ion mode with the potential between the spray needle and the orifice 4, 5 kV, nebulizer pressure of 0.4 bar, and a drying gas flow rate of 4 L/min at 200 °C. The sample flow rate was 180 L/min. Ionization mass spectra were collected at the ranges *m*/*z* 150–3000. The instrument was calibrated with an Agilent electrospray calibration solution (ESI-L low concentration Tuning Mix-Agilent Technologies, Agilent Product Number: G1969-85000) that was diluted with acetonitrile. The fluorescence measurements were performed on a fluorimeter Varian Cary Eclipse, (Varian, Palo Alto, CA, USA) equipped with 1.0 cm quartz cells and a thermostat bath. Cyclooxygenases (COX-1 and COX-2) activity experiments were carried out using a UV-Visible spectrophotometer Varian Cary 100 Bio (Varian, Palo Alto, CA, USA)

### 3.2. Materials

#### 3.2.1. Xanthohumol (**1**)

Xanthohumol (3’-[3”,3”-dimethylallyl]-2’,4’,4-trihydroxy-6’-methoxychalcone) (**1**) was isolated from supercritical carbon dioxide extracted hops (“Marynka”, crop 2011), obtained from The New Chemical Synthesis Institute in Puławy, Poland. Spent hops were immersed in ethyl acetate and extracted for 2 h at room temperature. The obtained extract was filtered and the solvent was evaporated off. The residue was chromatographed over Sephadex LH-20 with methanol as eluent. The fractions containing xanthohumol were collected, concentrated in vaccuo and purified by column chromatography on silica gel using chloroform:methanol (20:1 *v*/*v*) as the eluent. The obtained crude product was purified by column chromatography on silica gel using methylene chloride:diethyl ether:hexane:formic acid (200:40:28:1 *v*/*v*) as the eluent to give pure xanthohumol (**1**) (purity > 98%) as yellow-orange crystals. Spectral data of **1** were in agreement with the literature [58].

*Xanthohumol* (**1**): Yellow-orange crystals. ^1^H-NMR (600 MHz, DMSO-*d*_6_) δ*_H_*: 1.61 (3H, s, H-5”), 1.70 (3H, s, H-4”), 3.13 (1H, d, *J* = 7.1 Hz, H-1”), 3.87 (3H, s, C6’O-CH_3_), 5.14 (1H, m, H-2”), 6.08 (1H, s, H-5’), 6.84 (1H, m, H-3 and H-5), 7.58 (1H, m, H-2 and H-6), 7.67 (1H, d, *J* = 15.6 Hz, H-β), 7.77 (1H, d, *J* = 15.6 Hz, H-α), and 14.69 (C2’-OH). ^13^C-NMR (151 MHz, DMSO-*d*_6_) δ*_C_*: 17.7 (C-4”), 21.1 (C-1”), 25.5 (C-5”), 55.8 (C6’O-CH_3_), 91.0 (C-5’), 104.6 (C-1’), 107.4 (C-3’), 116.0 (C-3 and C-5), 123.1 (C-2”), 123.8 (C-α), 126.1 (C-1), 130.0 (C-3”), 130.5 (C-2 and C-6), 142.6 (C-β), 160.0 (C-4), 160.6 (C-6’), 162.4 (C-4’), 164.7 (C-2’), and 191.7 (C=O); HR ESI-MS: *m/z* calculated for C_21_H_22_O_5_ − H ([M − H]^−^): 353.1395. Found 353.1414 [M − H]^−^. UV (MeOH) λ_max_: 368.1 nm.

#### 3.2.2. α,β-Dihydroxanthohumol (**2**)

α,β-Dihydroxanthohumol (**2**) was prepared from xanthohumol (**1**) according to the procedure of regioselective hydrogenation described by Popłoński et al. [34]. Briefly, xanthohumol (**1**) (354.4 mg, 1 mmol) was dissolved in 25 mL of methanol in 100 mL two-neck round bottom flask, then 350 mg of 5% palladium on activated charcoal was added and the air dissolved in mixture was removed by bubbling with N_2_ for 20 min. The reaction mixture was flushed with H_2_ gas (1 atm, ballon) and stirred for 15 min at room temperature. Then the vessel was purged with N_2_ and the crude reaction mixture was filtrated and rinsed with methanol (three times × 10 mL). Obtained filtrate was evaporated and purified by column chromatography on silica gel 60 with a mixture of hexane:acetone (1:1 *v*/*v*) as the eluent. Using this procedure 301 mg (yield 85%) of α,β-dihydroxanthohumol (**2**) with over 98% of purity (according to HPLC) was obtained.

*α,β-Dihydroxanthohumol* (**2**): Pale yellow crystals, 85% (301 mg). ^1^H-NMR (600 MHz, CD_3_OD) δ*_H_*: 1.64 (3H, s, H-5”), 1.74 (3H, s, H-4”), 2.83 (2H, m, H-β), 3.20 (2H, m, H-1”, overlapped on H-α), 3.20 (2H, m, H-α, overlapped on H-1”), 3.82 (3H, s, C6’-OCH_3_), 5.18 (1H, m, H-2”), 5.98 (1H, s, H-5’), 6.70 (2H, m, *J* = 8.6 Hz, H-3, H-5), 7.03(2H, m, *J* = 8.6 Hz, H-2, H-6). ^13^C-NMR (151 MHz, CD_3_OD) δ*_C_*: 17.8 (C-4”), 22.2 (C-1”), 26.0 (C-5”), 31.6 (H-β), 47.5 (H-α), 55.9 (C6’-OCH_3_), 91.2 (C-5’), 105.8 (C-1’), 109.2 (C-3’), 116.1 (C-3, C-5), 124.2 (C-2”), 130.3 (C-2, C-6), 131.3 (C-3”), 133.9 (C-1), 156.5 (C-4), 162.6 (C-6’), 163.6 (C-4’), 165.7 (C-2’), 206.0 (C=O);UV (MeOH) λ_max_: 292.7 nm.

#### 3.2.3. (*Z*)-6,4’-Dihydroxy-4-methoxy-7-prenylaurone (**3**)

(*Z*)-6,4’-Dihydroxy-4-methoxy-7-prenylaurone (**3**) was prepared from xanthohumol (**1**) (Figure 1) according to the procedure of cyclization of 2’-hydroxychalcones to *Z*-aurones described by Venkateswarlu et al. [36]. Xanthohumol (**1**) (354.4 mg, 1 mmol) was dissolved in 10 mL of a solution of mercury acetate (319 mg, 1 mmol) in pyridine, at room temperature. Then the mixture was refluxed for 2 h and after cooling down, poured into ice-cold water (70 mL) and acidified with ice-cold 10% HCl. The mixture was extracted three times with ice-cold ethyl acetate. The combined ethyl acetate fractions were washed with ice-cold NaHCO_3_ solution (three times), and ice-cold distilled water (three times), then dried over MgSO_4_, filtered and evaporated under vacuum to give 423 mg of crude *Z*-aurone (**3**). The product was purified by column chromatography on silica gel 60 with a mixture of methylene chloride:acetonitrile:formic acid (140:25:1 *v*/*v*) as the eluent. Using this procedure 228.3 mg of (*Z*)-6,4’-dihydroxy-4-methoxy-7-prenylaurone (**2**) was obtained (yield 64.8%) with over 98% of purity (according to HPLC). To avoid light-induced isomerization of *Z*-aurones to *E*-aurones [35,37] all the procedures of synthesis and purification were performed in the darkness.

*(Z)-6,4’-Dihydroxy-4-methoxy-7-prenylaurone* (**3**): Orange crystals, yield 64.8% (228.3 mg). ^1^H-NMR (300 MHz, DMSO-*d*_6_) δ*_H_*: 1.65 (3H, s, H-5”), 1.80 (3H, s, H-4”), 3.33 (2H, m H-1”), 3.81 (3H, s, C4O-CH_3_), 5.25 (1H, m, H-2”), 6.23 (1H, s, H-5), 6.58 (1H, s, H-β), 6.85 (2H, m, H-3’and H-5’), and 7.78 (2H, m, H-2’ and H-6’). ^13^C-NMR (75 MHz, DMSO-*d*_6_) δ*_C_*: 17.6 (C-4’’), 21.2 (C-1”), 25.5 (C-5”), 55.6 (C4O-CH3), 94.0 (C-5), 103.0 (C-9), 103.9 (C-7), 109.5 (C-β), 115.9 (C-3’ and C-5’), 122.0 (C-2”), 123.4 (C-1’), 131.0 (C-3”), 132.8 (C-2” and C-6”), 146.0 (C-2), 157.0 (C-4), 158.9 (C-4’), 164.8 (C-8), 165.2 (C-6), and 179.0 (C=O). HR ESI-MS: *m/z* calculated for C_21_H_20_O_5_ − H: 351.1238 ([M − H]^−^). Found 351.1258 [M − H]^−^. UV (MeOH) λ_max_: 337.1, 399.6 nm.

Xanthohumol (**1**), α,β-dihydroxanthohumol (**2**) and (*Z*)-6,4’-dihydroxy-4-methoxy-7-prenylaurone (**3**) were used as a substrates for microbial glycosylation.

### 3.3. Biotransformation Products

*Xanthohumol 4’-O-β-d-glucopyranoside* (**4**) was obtained by means of microbial transformation of xanthohumol (**1**) by *Absidia coerulea* AM93 after 9-day biotransformation with yield 29.0% and by *Rhizopus nigricans* UPF701 after 14-day biotransformation with lower yield 14.2% [12].

*Xanthohumol 4’-O-β-d-(4’’’-O-methyl)glucopyranoside* (**5**) was obtained by microbial transformations of xanthohumol (**1**) by *Beauveria bassiana* AM278 after 6-day biotransformation with yield 24.7% and by *B. bassiana* AM446 after 3-day biotransformation with yield 23.0%. The method of obtaining both xanthohumol glucosides (**4** and **5**) was described previously [12,27].

*α,β-Dihydroxanthohumol 4’-O-β-d-glucopyranoside* (**6**) was obtained by means of microbial transformation of α,β-dihydroxanthohumol (**2**) by *Absidia coerulea* AM93 after 10-day biotransformation with yield 70.0% (122.2 mg). Pale yellow crystals, ^1^H-NMR (600 MHz, Acetone-*d*_6_) δ*_H_*: 1.62 (3H, s, H-5’’), 1.76 (3H, s, H-4’’), 2.86 (2H, m, H-β), 3.22 (1H, m, H-1’’a) 3.28 (2H, m, H-α), 3.40 (2H, m, H-1’’b overlapped on H-4’’’), 3.40 (2H, m, H-4’’’ overlapped on H-1’’b), 3.54 (1H, m, H-3’’’), 3.57 (1H, m, H-2’’’), 3.61 (1H, m, H-5’’’), 3.67 (1H, m, H-6’’’a), 3.93 (3H, s, C6’-OCH_3_), 3.94 (1H, m, H-6’’’b), 5.09 (1H, d, *J* = 7.4 Hz, H-1’’’), 5.24 (1H, m, H-2’’), 6.46 (1H, s, H-5’), 6.75 (2H, m, *J* = 8.4 Hz, H-3, H-5), 7.08 (2H, m, *J* = 8.4 Hz, H-2, H-6), 8.14 (C4-OH), 14.05 (C2’-OH). ^13^C-NMR (151 MHz Acetone-*d*_6_) δ*_C_*: 18.0 (C-4’’), 22.1 (C-1’’), 25.9 (C-5’’), 30.6 (C-β), 47.2 (C-α), 56.2 (C6’-OCH_3_), 62.8 (C-6’’’), 71.5 (C-4‘’’), 74.6 (C-2’’’), 78.3 (3’’’), 78.3 (C-5’’’), 91.1 (C-5’), 101.5 (C-1’’’), 106.9 (C-1’), 110.9 (C-3’), 116.0 (C-3, C-5), 124.0 (C-2’’), 130.2 (C-2, C-6), 131.0 (C-3’’), 133.2 (C-1), 156.4 (C-4), 162.2 (C-6’), 162.4 (C-4’), 164.4 (C-2’), 206,1 (C=O); HR ESI-MS: *m/z* calculated for C_27_H_34_O_10_ − H ([M − H]^−^): 517.2077. Found 517.2079 [M − H]^−^ UV (MeOH) λ_max_ 285.6 nm.

*α,β-Dihydroxanthohumol 4’-O-β-d-(4’’’-O-methyl)glucopyranoside* (**7**) was obtained in biotransformation catalyzed by *Beauveria bassiana* AM278 after 10-day biotransformation with yield 39.0% (69.9 mg). Pale yellow crystals, ^1^H-NMR (600 MHz, Acetone-*d*_6_) δ*_H_*: 1.62 (3H, s, H-4”), 1.76 (3H, s, H-5”), 2.86 (2H, m, H-β), 3.13 (1H, m, H-4’’’), 3.22 (1H, dd, *J* = 13.8 H and 6.9 Hz, H-1’’a), 3.28 (2H, m, H-α), 3.40 (1H, dd, *J* = 13.8 H and 7.8 Hz, H-1”b), 3.55 (3H, s, C4’’’-OCH_3_), 3.57 (1H, m, H-3’’’, overlapped on H-2’’’), 3.58 (1H, m, H-2’’’, overlapped on H-3’’’), 3.63 (1H, m, H-5’’’), 3.68 (1H, m, H-6’’’a), 3.87 (1H, m, H-6’’’b), 3.91 (3H, s, C6’-OCH_3_), 5.06 (1H, d, *J* = 7.7 Hz, H-1’’’), 5.24 (1H, m, H-2”), 6.34 (1H, s, H-5’), 6.75 (2H, m, *J* = 8.3 Hz, H-3, H-5), 7.08 (2H, m, *J* = 8.3 Hz, H-2, H-6). ^13^C-NMR (151 MHz Acetone-*d*_6_) δ*_C_*: 17.7 (C-5”), 22.1 (C-1”), 25.9 (C-4”), 30.6 (C-β), 47.1 (C-α), 56.1 (C6’-OCH3), 60.6 (C4’’’-OCH_3_), 62.3 (C-6’’’), 74.9 (C-2’’’), 77.5 (C-3’’’), 78.3 (C-5’’’), 80.6 (C-4’’’), 91.1 (C-5’), 101.2 (C-1’’’), 106.9 (C-1’), 111.0 (C-3’), 116.0 (C-3, C-5), 123.9 (C-2”), 130.2 (C-2, C-6), 131.0 (C-3”), 133.2 (C-1), 156.4 (C-4), 162.2 (C-6’), 162.3 (C-4’), 164.4 (C-2’), 206.3 (C=O); HR ESI-MS *m/z* calculated for C_28_H_36_O_10_-H ([M − H]^−^): 531.2236. Found 531.2237 [M − H]^−^. UV (MeOH) λ_max_ 285.6 nm.

*(Z)-6,4’-Dihydroxy-4-methoxy-7-prenylaurone 6-O-β-d-glucopyranoside* (**8**) was obtained by means of microbial transformation of (*Z*)-6,4’-dihydroxy-4-methoxy-7-prenylaurone (**3**) by *Absidia glauca* AM177 after 9-day biotransformation with yield 47.9% (56.0 mg). Orange crystals, ^1^H-NMR (600 MHz, CD_3_OD) δ*_H_*: 1.70 (3H, s, H-5”), 1.87 (3H, s, H-4”), 3.36 (1H, m, H-4’’’), 3.44 (1H, dd, *J* = 14.8, and 7.2 Hz, H-1”a), 3.52 (1H, m, H-3’’’, overlapped on H-5’’’), 3.52 (1H, m, H-5’’’ overlapped on H-3’’’), 3.55 (1H, m, H-2’’’, overlapped on H-1”b), 3.56 (1H, m, H-1”b, overlapped on H-2’’’), 3.68 (1H, dd, *J* = 11.9, and 6,6 Hz, H-6’’’a), 3.94 (3H, s, C4-OCH_3_ overlapped on H-6’’’b), 3.96 (1H, m, H-6’’’b overlapped on C4-OCH_3_), 5.08 (1H, d, *J* = 7.6 Hz, H-1’’’), 5.33 (1H, m, H-2”), 6.60 (1H, s, H-5), 6.68 (1H, s, H-β), 6.85 (2H, m, H-3’, H-5’), 7.78 (2H, m, H-2’, H-6’); ^13^C-NMR (151 MHz, CD_3_OD) δ*_C_*: 18.2 (C-4”), 22.6 (C-1”), 25.9 (C-5”), 56.6 (C4-OCH_3_), 62.7 (C-6’’’), 71.5 (C-4’’’), 74.9 (C-2’’’), 78.4 (C-3’’’), 78.8 (C-5’’’), 94.9 (C-5), 102.1 (C-1’’’), 106.7 (C-7), 108.5 (C-7), 113.4 (C-β), 117.0 (C- 3’, C-5’), 123.1 (C-2”), 125.1 (C-1’), 132.7 (C-3”), 134.5 (C-2”,C-6”), 147.6 (C-2), 159.0 (C-4’), 160.8 (C-4), 165.5 (C-8), 166.4 (C-6), 183.1 (C=O); HR ESI-MS *m/z* calculated for C_27_H_30_O_10_Na + H ([M + H]^+^): 537.1731. Found 515. 537.1740 [M + H]^+^. UV (MeOH) λ_max_: 334.6, 405.3 nm.

*(Z)-6,4’-Dihydroxy-4-methoxy-7-prenylaurone 6-O-β-d-(4’’’-O-methyl)glucopyranoside* (**9**) was obtained in biotransformation of of (*Z*)-6,4’-dihydroxy-4-methoxy-7-prenylaurone (**3**) catalyzed by *Beauveria bassiana* AM278 after 18-day biotransformation with yield 20.0% (24.0 mg). Orange crystals, ^1^H-NMR (300 MHz, DMSO-*d*_6_) δ*_H_*: 1.65 (3H, s, H-5”), 1.81 (3H, s, H-4”), 3.00 (1H, t, *J* = 8.9 Hz, H-5’’’), 3.36 (1H, m, H-1”a, overlapped on H-2’’’), 3.36 (1H, m, H-2’’’, overlapped on H-1”a), 3.44 (1H, m, H-3’’’, overlapped on C4’’’-OCH_3_ and H-4’’’), 3.46 (3H, s, C4’’’-OCH_3_, overlapped on H-3’’’ and H-4’’’), 3.49 (1H, m, H-4’’’, overlapped on H-3’’’ and C4’’’-OCH_3_), 3.53 (1H, m, H-1” b, overlapped on H-6’’’a), 3.53 (1H, m, H-6’’’a, overlapped on H-1”b), 3.67 (1H, m, H-6’’’b), 3.87 (3H, s, C4-OCH_3_), 5.08 (1H, d, *J* = 7.5 Hz, H-1’’’), 5.29 (1H, m, H-2”), 6.55 (1H, H-5), 6.64 (1H, s, H-β), 6.86 (2H, m, H-3’, H-5’), 7.79 (2H, m, H-2’, H-6’); ^13^C-NMR (75 MHz, DMSO-*d*_6_) δ*_C_*: 17.7 (C-4”), 21.3 (C-1”), 25.5 (C-5”), 55.9 (C4-OCH_3_), 59.7 (C-4’’’-OCH_3_), 60.4 (C-6’’’), 73.5 (C-2’’’), 76.1 (C-4’’’), 76.5 (C-3’’’), 79.4 (C-5’’’), 93.9 (C-5), 100.3 (C-1’’’), 104.7 (C-7), 106.1 (C-9), 110.5 (C-β), 116.1 (C-3’, C-5’), 122.0 (C-2’’), 122.8 (C-1’), 131.0 (C-3”), 133.0 (C-2’,C-6’), 145.5 (C-2), 156.9 (C-4’), 159.7 (C-4), 163.2 (C-8), 164.2 (C-6), 179.5 (C=O); HR ESI-MS *m/z* calculated for C_28_H_32_O_10_ + H ([M + H]^+^): 529.2068. Found 529.2077 [M + H]^+^. UV (MeOH) λ_max_: 333.4, 405.3 nm.

### 3.4. Microorganisms

Fungal strains used for biotransformations were purchased from Institute of Biology and Botany of the Wrocław Medical University, Poland (indexed AM) and from Department of Plant Protection of Wrocław University of Environmental and Life Sciences, Poland (indexed UPF). Microorganisms were maintained on agar slants at 5 °C and grown on a Sabouraud medium (3% glucose and 1% peptone) at 25 °C.

### 3.5. Conditions for Biotransformations

The cultures were shaken on rotatory shakers (130 speed, 7 amplitude) at 28 °C in 300 mL Erlenmeyer flasks with 100 mL of the Sabouraud medium. Agar slant cultures were used to obtain the preculture (100 mL Erlenmeyer flasks with 30 mL of the medium) and then 3-day precultures were transferred to the main cultures media (5 mL to the 100 mL). Substrates were added after six days of culturing. One hundred twenty milligrams of substrate **2** or 80 mg of substrate **3** dissolved in 4 mL of DMSO were equally distributed among four flasks (1 mL each). The reactions were carried until the substrate was metabolized (the progress of conversion was monitored by HPLC). Then the cultures were acidified with 1 M HCl to pH around 5 (if necessary) and reaction mixtures were extracted with ethyl acetate (three times × 40 mL). Obtained extracts were dried over anhydrous magnesium sulphate, filtered and evaporated under vacuum. Substrate stability control consisted of the particular substrate dissolved in DMSO and a sterile growth medium incubated without microorganisms. To avoid light-induced isomerization of *Z*-aurones to *E*-aurones in case of substrate **3** all procedures (biotransformation, extraction, purification of crude products etc.) were carried out in darkness.

### 3.6. Products Isolation

The products of biotransformations were separated by column chromatography on silica gel 60 (230–400 mesh, Merck) using chloroform:methanol (9:1 *v*/*v*) for compounds **6** and **7** or chloroform:acetonitryle:formic acid (70:10:1 *v*/*v*) for compounds **8** and **9** as developing solvents. Product structures were elucidated by NMR and MS spectroscopy methods.

### 3.7. Methods

#### 3.7.1. Fluorescence Quenching of Human Serum Albumin

Analysis of the potential interaction of studied compounds with human serum albumin (HSA) was performed according to the work of Strugała et al. [59] with minor modifications. The fluorescence measurements were performed on a fluorimeter (Cary Eclipse, Varian, Palo Alto, CA, USA) equipped with 1.0 cm quartz cells and a thermostat bath. All quenching experiments were performed at 300, 305, 310 and 315 K temperature. The final concentration of HSA essentially fatty acid free solutions was 1.5 × 10^−5^ M in a phosphate buffer of pH 7.4. The excitation wavelength was set at 280 nm (excitation of the Trp and Tyr), and the emission spectra were read at 285–460 nm. The excitation and emission slits were both set to 5 nm. Our method consisted of tracing the quenching of natural HSA fluorescence caused by all compounds (the final concentrations 1, 3, 5, 7, 9, 11, 13 and 15 µM) added successively. The stock solution of all compounds was freshly prepared by dissolving in DMSO. The final amount of DMSO was always less than 2%, and it has been verified that such amounts of the solvent do not affect the fluorescence of HSA. Each experiment was performed in three independent replicates (*n* = 3).

#### 3.7.2. Cyclooxygenase (COX-1 and COX-2) Activity

The anti-inflammatory activity of the tested compounds was studied according to the procedure described in detail by Strugała et al. [60]. In short, to a cuvette containing Tris-HCl buffer (pH 8.0) the following were successively added: tested compound, hematin (0.1026 mM) and cyclooxygenases (COX-1 or COX-2—independent experiments) at 1 mg/mL. After mixing and incubation, TMPD was added at 24.35 mM. To initiate the reaction, arachidonic acid was added at a concentration of 35 mM. Reactions were carried out in 10 mm path-length quartz cuvettes and a final volume of the sample was 1 mL. Changes in absorbance of the sample were followed for 3 min, using a spectrophotometer at a wavelength of λ = 611 nm (Cary 100 Bio, Varian, Palo Alto, CA, USA). The measurements were performed at room temperature. The control sample contained the appropriate amount of DMSO instead of studied compound. At least three independent experiments were performed. The percentage of inhibition was calculated using the following formula:

(5) $$\frac{A_{control-}A_{sample}}{A_{control}}\cdot 100\%$$

where *A_control_* is the absorbance of the control probe and *A_sample_* is the absorbance of the probe with tested compound. IC_50_ values were calculated for each independent experiment separately.

## 4. Conclusions

Regioselective glycosylation of biologically active flavonoid aglycones catalyzed by microorganisms is an interesting and desired reaction, which significantly increases the water solubility of the compound and, therefore, may improve bioavailability of flavonoids. The selection of fungi such as *Absidia* and *Beauveria bassiana*, which exhibit high regioselectivity of glycosylation and low substrate specificity, gives opportunity for obtaining numerous desired glycosides in sufficient amounts for biological assays. Prenylated flavonoid xanthohumol (**1**) has a strong therapeutic potential, thus it is a very good starting material for obtaining other biologically active molecules. It is believed that the presence of sugar moiety in flavonoid molecule is a fundamental determinant of their absorption in humans. Deposition, transportation, metabolism and efficacy of drugs are strongly affected by their binding to human serum albumin [59,60,61]. Therefore, the studies on interactions of biologically active aglycones and their glycosylated derivatives with plasma proteins have an important role in understanding the action mechanism, pharmacokinetics and toxicity of these compounds. Two biologically active prenylated aglycones **2**, **3** belonging to different flavonoid classes were obtained from xanthohumol (**1**). The method of synthesis (*Z*)-6,4’-dihydroxy-4-methoxy-7-prenylaurone (**3**) proposed by us (with yield close to 65%) is the most efficient published so far. The aglycones **1**–**3** were used as substrates for fungal biotransformation. As a result of biotransformation, six glycosylated flavonoids were obtained (**4**–**9**). To our best knowledge, four glycosides (**6**–**9**) have never been published so far. Binding of all of the compounds to human serum albumin and inhibition of pro-inflammation enzymes COX-1 and COX-2 activities were investigated.

The binding affinity to HSA in case of tested aglycones strongly depends on the structure of molecule skeleton. The values of this parameter are as follows: chalcone **1** > dihydrochalcone **2** > aurone **3**. This proves that, in the case of prenylated flavonoids, the type of flavonoids skeleton has a great importance in binding to human plasma proteins. The presence of large polar sugar group significantly decreased binding affinity to HSA. Sugar moiety generates steric hindrance in the binding pocket and by increasing polarity of whole molecule, lessens the ability of the flavonoid glycosides to penetrate into the Trp-rich hydrophobic interior regions of HSA. Similar trend was observed in studies on inhibition of cyclooxygenases activity. Chalcone **1** was the strongest anti-inflammatory agent. It was two-fold stronger inhibitor than two other tested aglycones (**2**–**3**). Glycosylation of prenylated flavonoids caused the decrease of their activity 3–4 fold in the case of aglycones **2** and **3** and close to six fold in case of glycosides **4** and **5** compared to xanthohumol (**1**). Our studies showed that modification of structure of aglycone (**1**–**3**) by conjugation with β-glucopyranose (compounds **4**, **6**, and **8**) or 4-*O*-methyl-β-glucopyranose (compounds **5**, **7**, and **9**) decreased affinity to human plasma proteins as well as inhibition of both cyclooxygenases. Presented results show that aglycones are significantly more active than their glycosides. However, poor solubility and low bioavailability of aglycones limits their therapeutic potential. Modification of structure by coupling with sugar moiety may enhance absorption in the small intestine, which was observed for quercetin and its 3-*O*-β-d-glucoside [62,63]. Only studies on biological activities of aglycones and their glycosides coupled with the studies on bioavailability and absorption from digestive tract will lead to an unambiguous answer to the question whether glycosylation of bioactive aglycones is desired.

## Figures and Tables

**Table 1:** Quenching (*K_sv_*) and binding (*K_b_*) constants and thermodynamic parameters (*n*, *ΔG*, *ΔH* and *ΔS*) of the tested compounds and human serum albumin at different temperatures.

Compound	*T(K)*	*K_sv_* (10^4^ M^−1^)	*K_q_* (10^12^ M^−1^ s^−1^)	*K_b_* (10^4^ M^−1^)	*n*	*∆G* (kJ/M)	*∆H* (kJ/M)	*∆S* (J/M/K)
**1**	300	12.224	24.448	8.624	0.971	−12.310	−21.601	−31.239
	305	12.087	24.174	5.462	0.935	−12.010		
	310	11.958	23.916	3.776	0.903	−11.794		
	315	11.759	23.518	3.300	0.893	−11.833		
**4**	300	7.488	14.976	3.271	0.931	−11.240	−12.536	−4.583
	305	7.180	14.360	2.207	0.899	−11.014		
	310	6.897	13.794	2.082	0.898	−11.125		
	315	6.326	12.652	1.776	0.906	−11.111		
**5**	300	8.256	16.512	0.455	0.748	−9.090	−54.307	−149.822
	305	8.069	16.138	0.364	0.733	−9.025		
	310	7.905	15.810	0.114	0.614	−7.858		
	315	7.582	15.164	0.043	0.515	−6.894		
**2**	300	8.806	17.612	3.635	0.928	−11.375	−12.315	−3.027
	305	8.733	17.466	3.463	0.925	−11.511		
	310	8.652	17.304	2.286	0.890	−11.235		
	315	8.441	16.882	2.247	0.881	−11.396		
**6**	300	4.062	8.124	0.448	0.808	−9.097	−21.593	−42.054
	305	4.034	8.068	0.245	0.755	−8.564		
	310	3.746	7.492	0.222	0.751	−8.593		
	315	3.575	7.150	0.159	0.727	−8.360		
**7**	300	3.618	7.236	0.611	0.908	−10.204	−10.501	−1.551
	305	3.474	6.948	0.566	0.860	−9.728		
	310	3.400	6.800	0.529	0.879	−10.114		
	315	3.099	6.198	0.478	0.868	−10.041		
**3**	300	9.356	18.712	1.425	0.838	−10.325	−9.933	−1.766
	305	9.255	18.510	1.364	0.832	−10.445		
	310	9.133	18.266	1.189	0.821	−10.439		
	315	8.907	17.814	1.047	0.812	−10.452		
**8**	300	1.796	3.592	0.485	0.929	−10.429	−43.061	−37.029
	305	1.751	3.502	0.302	0.881	−10.317		
	310	1.692	3.384	0.266	0.879	−10.493		
	315	1.611	3.222	0.224	0.802	−9.861		
**9**	300	2.495	4.990	0.915	0.909	−12.517	−44.673	−106.347
	305	2.374	4.748	0.645	0.879	−12.337		
	310	2.389	4.778	0.544	0.865	−12.266		
	315	2.242	4.484	0.581	0.674	−10.698		

Standard deviations (mean value of three independent experiments) were lower than 10%.

**Table 2:** Inhibition activity of tested compounds (**1**–**9**) against cyclooxygenase-1 (COX-1) and cyclooxygenase-2 (COX-2).

Compound	IC_50_^COX-1^ (µM)	IC_50_^COX-2^ (µM)
**1**	62.10 ± 3.48	51.86 ± 3.28
**4**	352.32 ± 10.91	302.95 ± 8.54
**5**	384.87 ±14.10	321.75 ±13.01
**2**	124.50 ± 7.61	103.8 ± 6.11
**6**	397.93 ± 7.51	370.81 ± 15.06
**7**	458.67 ± 4.02	451.18 ± 12.07
**3**	133.23 ± 6.91	109.02 ± 6.82
**8**	419.80 ± 8.34	405.01 ± 10.26
**9**	493.97 ± 6.34	482.16 ± 10.39

## Supplementary Materials

Supplementary materials can be found online.
