# Supplementary material for: The Influence of Glycosylation of Natural and Synthetic Prenylated Flavonoids on Binding to Human Serum Albumin and Inhibition of Cyclooxygenases COX-1 and COX-2
Source: Molecules. 2017 Jul 21;22(7):1230. doi: 10.3390/molecules22071230 (PMC6152009; doi:10.3390/molecules22071230)

Supplementary data

The influence of glycosylation of natural and synthetic prenylated flavonoids on binding to human serum albumin and inhibition of cyclooxygenases COX-1 and COX-2

Tomasz Tronina*[1], Paulina Strugała[2], Jarosław Popłoński[1], Aleksandra Włoch[2], Sandra Sordon[1], Agnieszka Bartmańska[1] and Ewa Huszcza[1]

[1]Department of Chemistry, Wrocław University of Environmental and Life Sciences, Norwida 25, 50-375 Wrocław, Poland.[2]Department of Physics and Biophysics, Wrocław University of Environmental and Life Sciences, Norwida 25, 50-375 Wrocław, Poland. *Corresponding Author: E-mail address: tomasz.tronina@upwr.edu.pl, Phone: +48 713205197; fax: +48 3207744

**Table of Contents:**	**Page**
*^{1}H NMR spectrum of α,β-dihydroxanthohumol (**2**)*	S2
*^{13}C NMR and DEPT 135° spectra of α,β-dihydroxanthohumol (**2**)*	S2
*UV spectra of xanthohumol (**1**) and (Z)-6,4'-dihydroxy-4-methoxy-7-prenylaurone (**3**)*	S3
*HRMS spectrum of (Z)-6,4'-dihydroxy-4-methoxy-7-prenylaurone (**3**)*	S3
*^{1}H NMR spectra of xanthohumol (**1**) and (Z)-6,4'-dihydroxy-4-methoxy-7-prenylaurone (**3**)*	S4
*Fragments of ^{1}H NMR spectra of xanthohumol (**1**) and (Z)-6,4'-dihydroxy-4-methoxy-7-prenylaurone (**3**)*	S5
*^{13}C NMR spectra of xanthohumol (**1**) and (Z)-6,4'-dihydroxy-4-methoxy-7-prenylaurone (**3**)*	S6
*^{1}H–^{1}H NMR (COSY) spectrum of (Z)-6,4'-dihydroxy-4-methoxy-7-prenylaurone (**3**)*	S7
*^{1}H– ^{13}C NMR (HSQC) spectrum of (Z)-6,4'-dihydroxy-4-methoxy-7-prenylaurone (**3**)*	S7
*UV spectrum of α,β-dihydroxanthohumol 4' -O-β-D-glucopyranoside (**6**)*	S8
*HRMS spectrum of α,β-dihydroxanthohumol 4' -O-β-D-glucopyranoside (**6**)*	S8
*^{1}H NMR and ^{13}C NMR spectra of α,β-dihydroxanthohumol 7-O-β-D-glucopyranoside (**6**)*	S9
*^{1}H NMR - ^{1}H NMR (COSY) spectrum of α,β-dihydroxanthohumol 7-O-β-D-glucopyranoside (**6**)*	S10
*^{1}H NMR - ^{13}C NMR (HSQC) spectrum of α,β-dihydroxanthohumol 7-O-β-D-glucopyranoside (**6**)*	S10
*UV spectrum of α,β-dihydroxanthohumol 7-O-β-D-(4'''-O-methyl)glucopyranoside (**7**)*	S11
*HRMS spectrum of α,β-dihydroxanthohumol 7-O-β-D-(4'''-O-methyl)glucopyranoside (**7**)*	S11
*^{1}H NMR and ^{13}C NMR spectra of α,β-dihydroxanthohumol 7-O-β-D-(4'''-O-methyl)glucopyranoside (**7**)*	S12
*^{1}H NMR - ^{1}H NMR (COSY) spectrum of α,β-dihydroxanthohumol 7-O-β-D-(4'''-O-methyl)glucopyranoside (**7**)*	S13
*^{1}H NMR - ^{13}C NMR (HSQC) spectrum of α,β-dihydroxanthohumol 7-O-β-D-(4'''-O-methyl)glucopyranoside (**7**)*	S13
*UV spectrum of (Z)-6,4'-dihydroxy-4-methoxy-7-prenylaurone 6-O-β-D-glucopyranoside (**8**)*	S14
*HRMS spectrum of (Z)-6,4'-dihydroxy-4-methoxy-7-prenylaurone 6-O-β-D-glucopyranoside (**8**)*	S14
*^{1}H NMR and ^{13}C NMR spectra of (Z)-6,4'-dihydroxy-4-methoxy-7-prenylaurone 6-O-β-D-glucopyranoside (**8**)*	S15
*^{1}H NMR - ^{1}H NMR (COSY) spectrum of (Z)-6,4'-dihydroxy-4-methoxy-7-prenylaurone 6-O-β-D-glucopyranoside (**8**)*	S16
*^{1}H NMR - ^{13}C NMR (HSQC)spectrum of (Z)-6,4'-dihydroxy-4-methoxy-7-prenylaurone 6-O-β-D-glucopyranoside (**8**)*	S16
*UV spectrum of (Z)-6,4'-dihydroxy-4-methoxy-7-prenylaurone 6-O-β-D-(4'''-O-methyl)glucopyranoside (**9**)*	S17
*HRMS spectrum of (Z)-6,4'-dihydroxy-4-methoxy-7-prenylaurone 6-O-β-D-(4'''-O-methyl)glucopyranoside (**9**)*	S17
*^{1}H NMR and ^{13}C NMR spectra of (Z)-6,4'-dihydroxy-4-methoxy-7-prenylaurone 6-O-β-D-(4'''-O-methyl)glucopyranoside (**9**)*	S18
*^{1}H NMR - ^{1}H NMR (COSY) spectrum of (Z)-6,4'-dihydroxy-4-methoxy-7-prenylaurone 6-O-β-D-(4'''-O-methyl)glucopyranoside (**9**)*	S19
*^{1}H NMR - ^{13}C NMR (HSQC) spectrum of (Z)-6,4'-dihydroxy-4-methoxy-7-prenylaurone 6-O-β-D-(4'''-O-methyl)glucopyranoside (**9**)*	S19

Figure S1. *^{1}H NMR spectrum of α,β-dihydroxanthohumol (**2**) (600 MHz, CD_3OD, Temp. 25 ºC)*

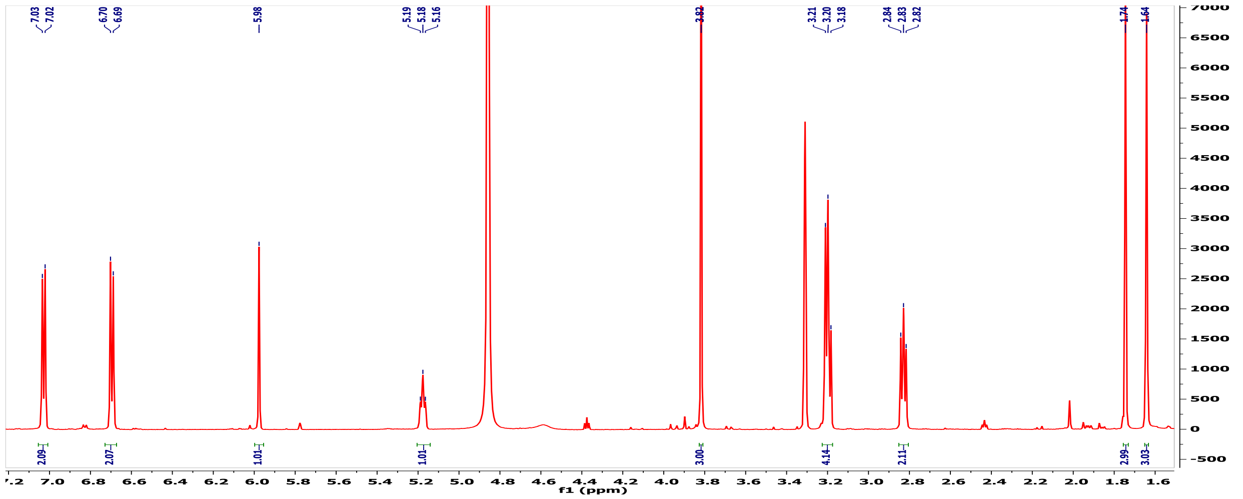

Figure S2. *^{13}C NMR and DEPT 135° spectra of α,β-dihydroxanthohumol (**2**) (151 MHz, CD_3OD, Temp. 25 ºC)*

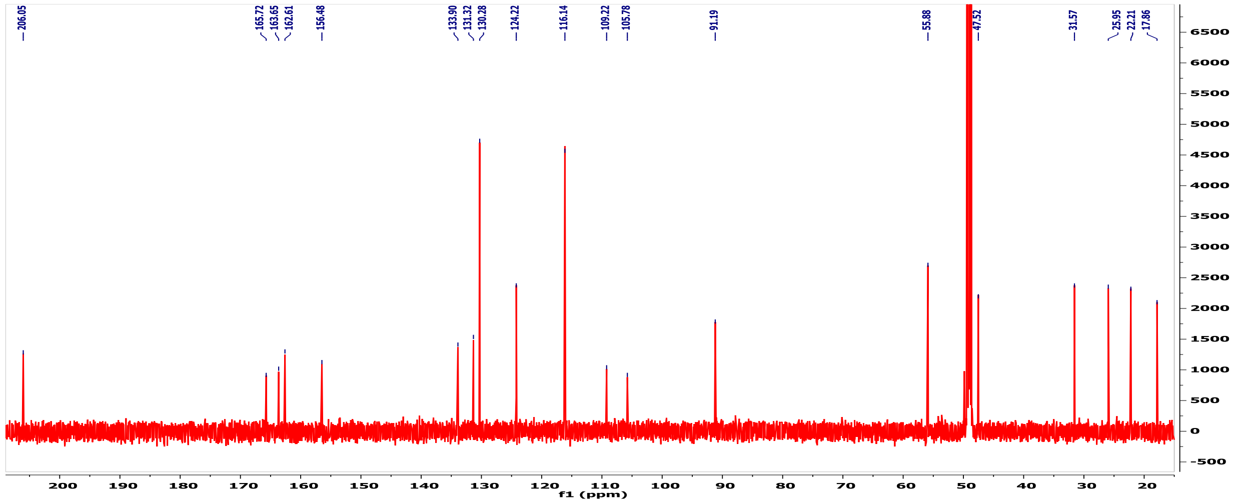

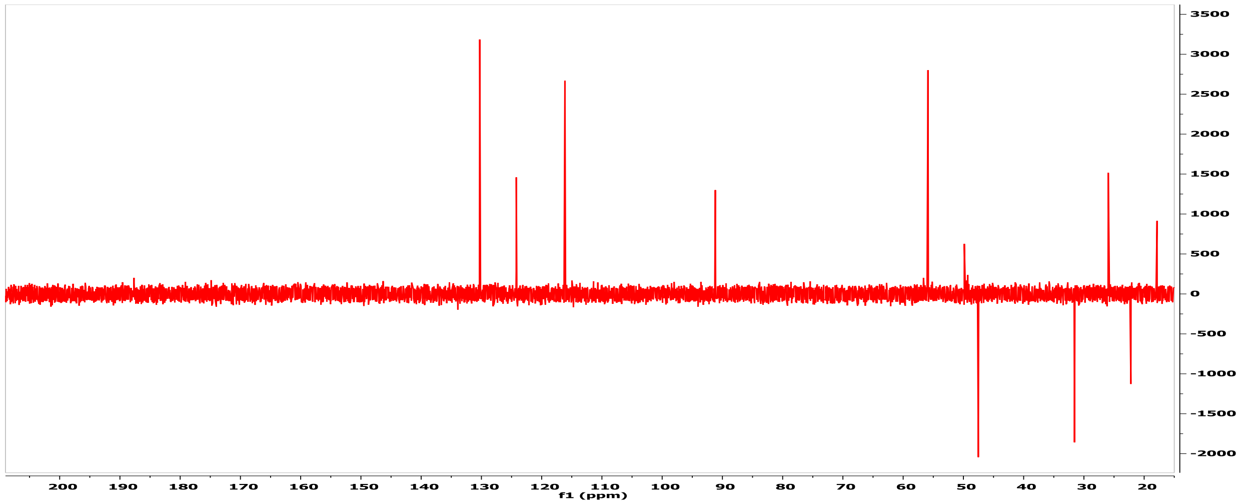

Figure S3: *UV spectra of:* ***A*** *xanthohumol (**1**),* ***B*** *(Z)-6,4'-dihydroxy-4-methoxy-7-prenylaurone (**3**) (MeOH, Temp. 20 °C)*

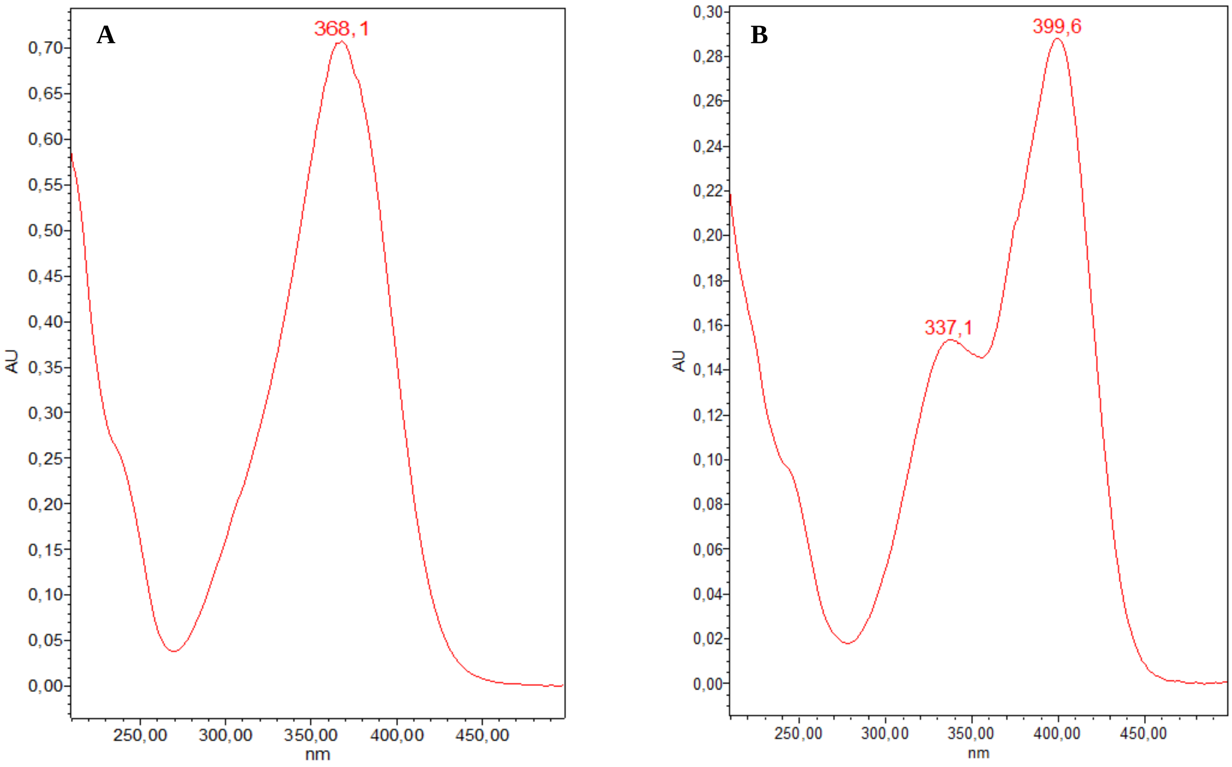

Figure S4. *HRMS spectrum of (Z)-6,4'-dihydroxy-4-methoxy-7-prenylaurone (**3**)*

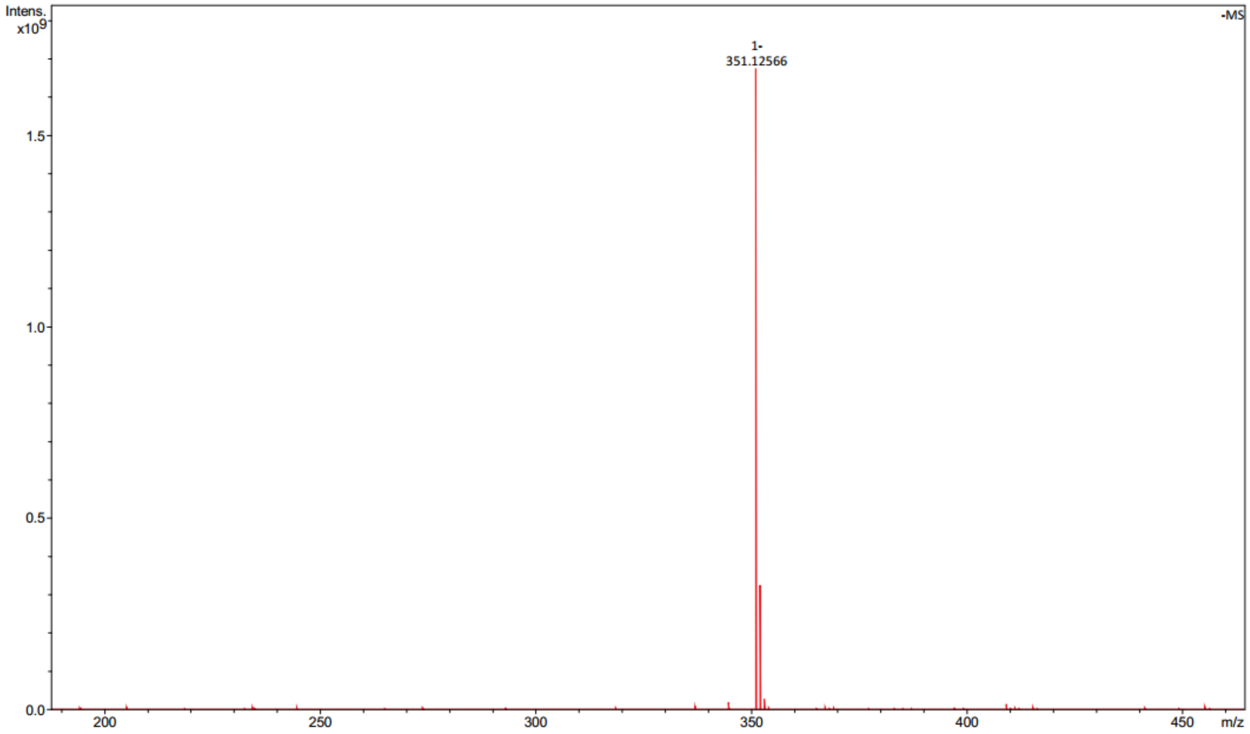

Figure S5. *^{1}H NMR spectra of:* **A** *xanthohumol (***1***) (600 MHz, DMSO-d$_6$),* **B** *(Z)-6,4'-dihydroxy-4-methoxy-7-prenylaurone (***3***) (300 MHz, DMSO-d$_6$, Temp. 25 ºC)*

A

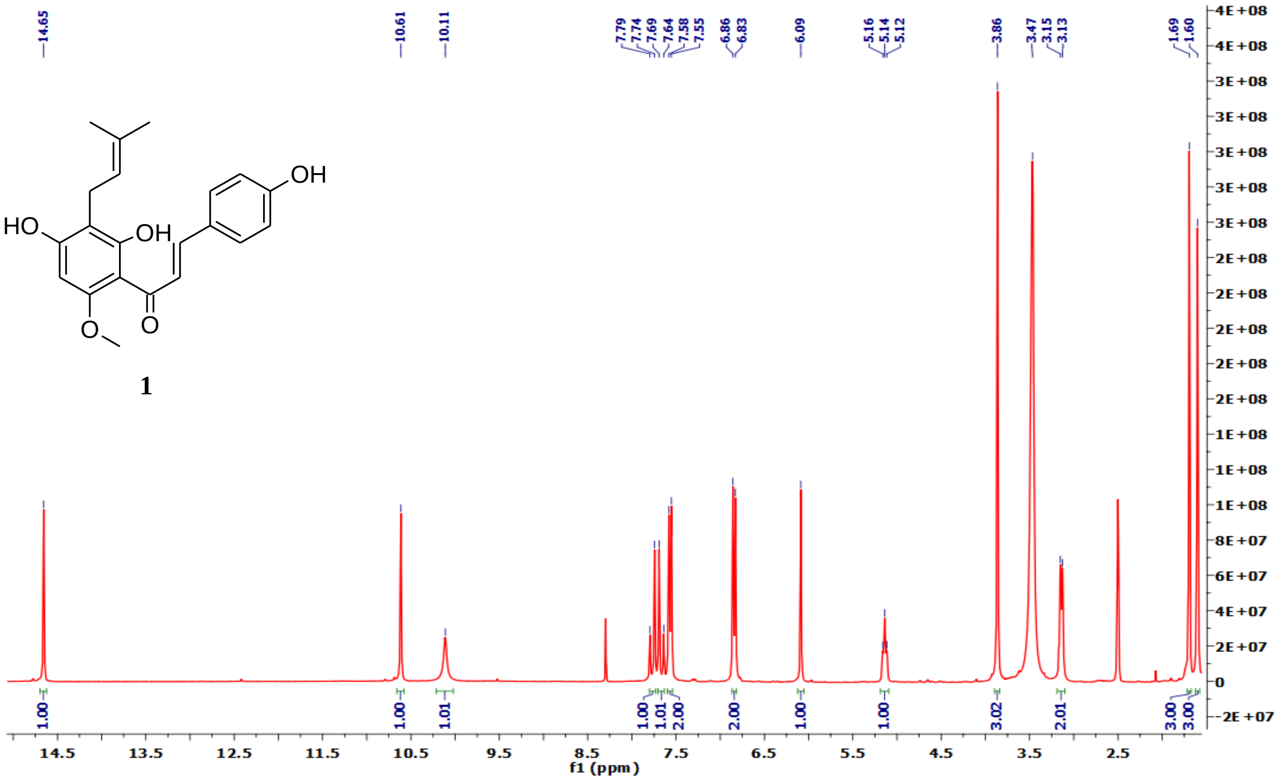

B

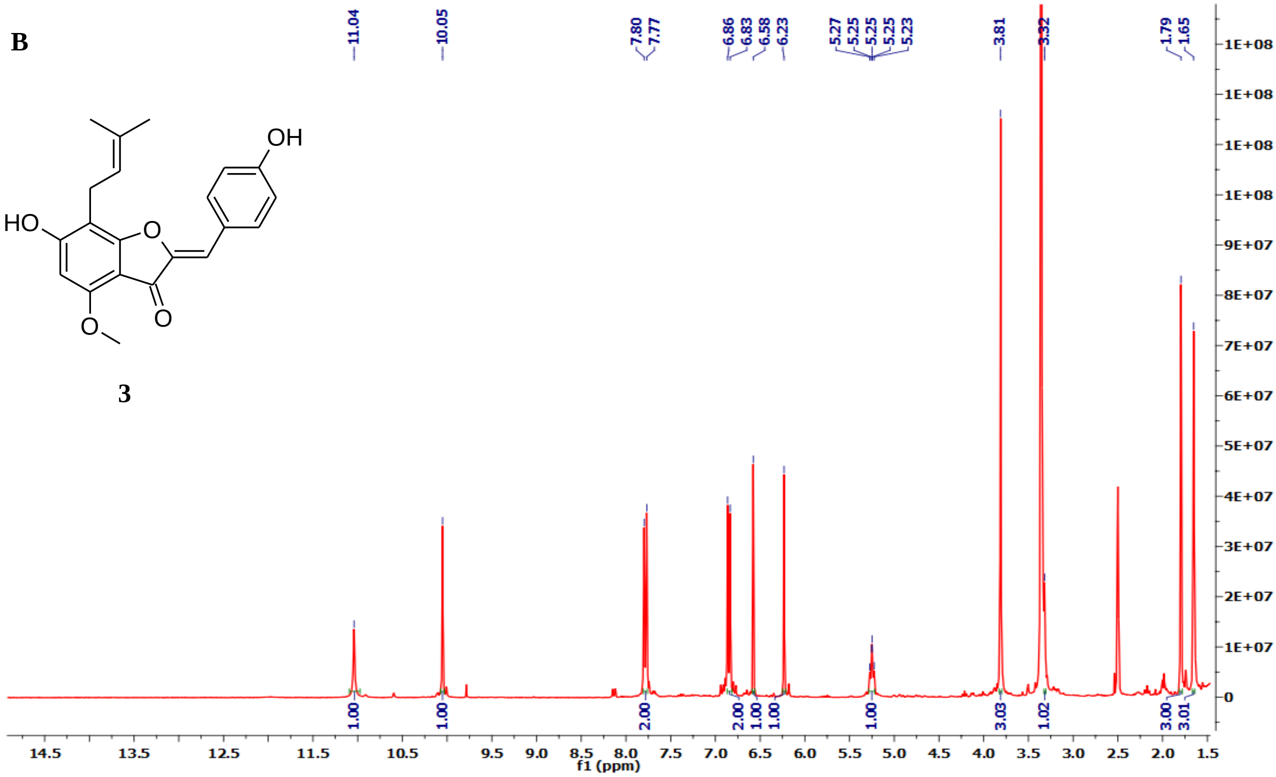

Figure S6. *Fragments of ^{1}H NMR spectra of:* ***A*** *xanthohumol (****1****) (600 MHz, DMSO-d₆),* ***B*** *aurone (****3****) (300 MHz, DMSO-d₆, Temp. 25 ºC)*

A

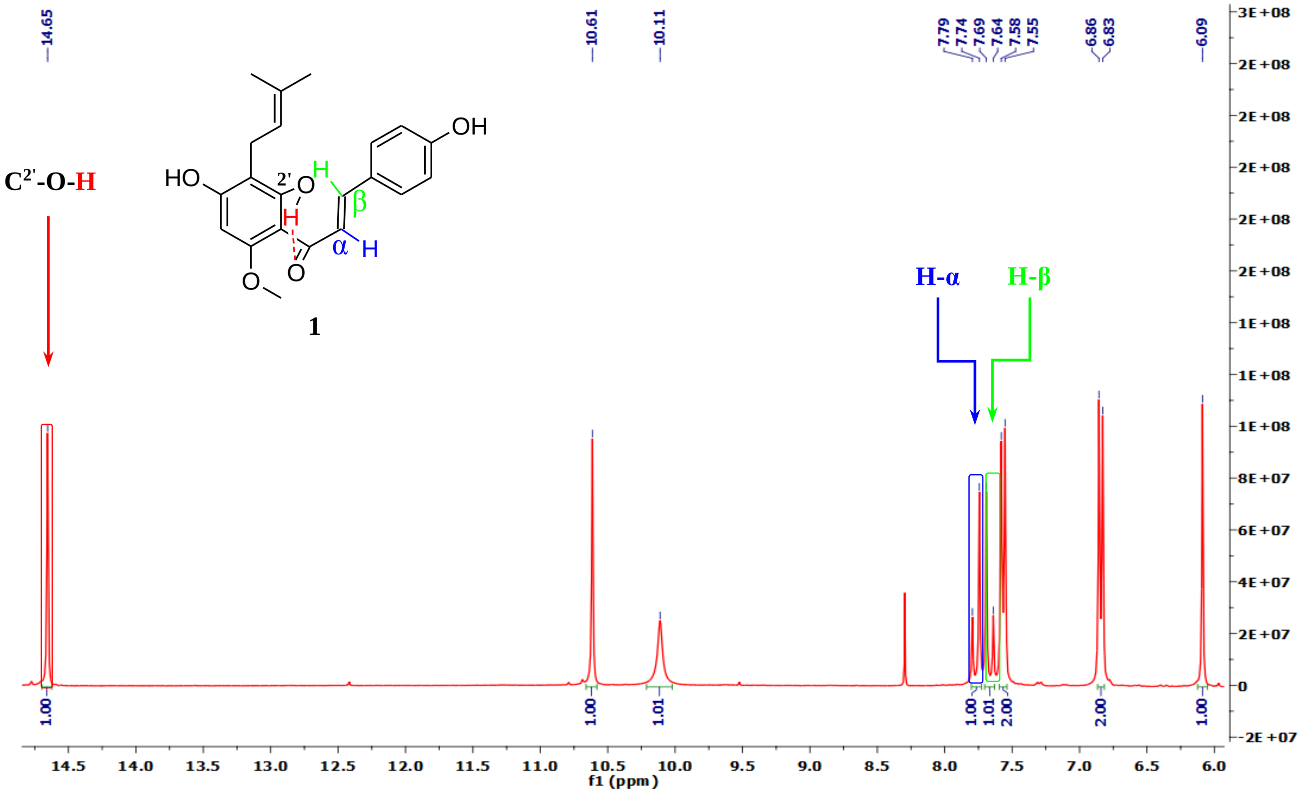

B

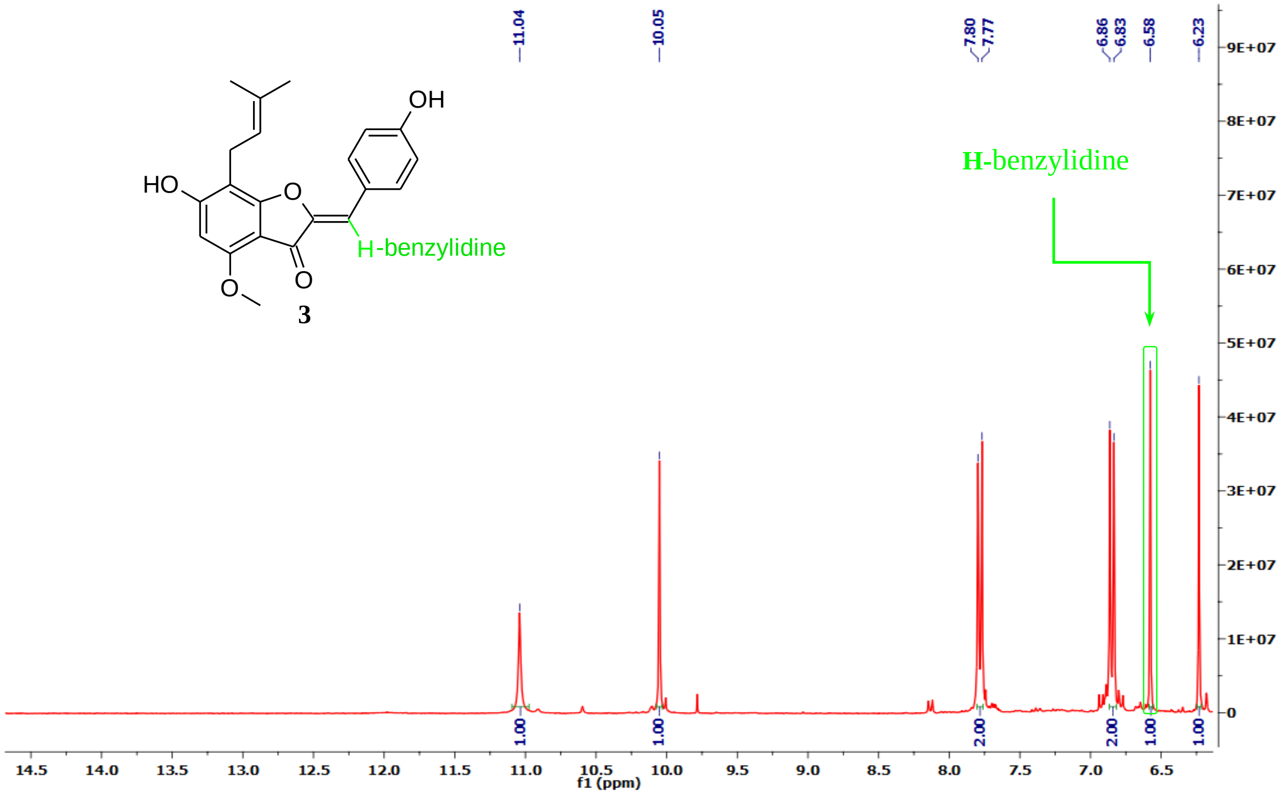

Figure S7. *^{13}C NMR spectra of:* ***A*** *xanthohumol (**1**) (151 MHz, DMSO-d$_6$),* ***B*** *auron (**3**) (75 MHz, DMSO-d$_6$, Temp. 25 ºC)*

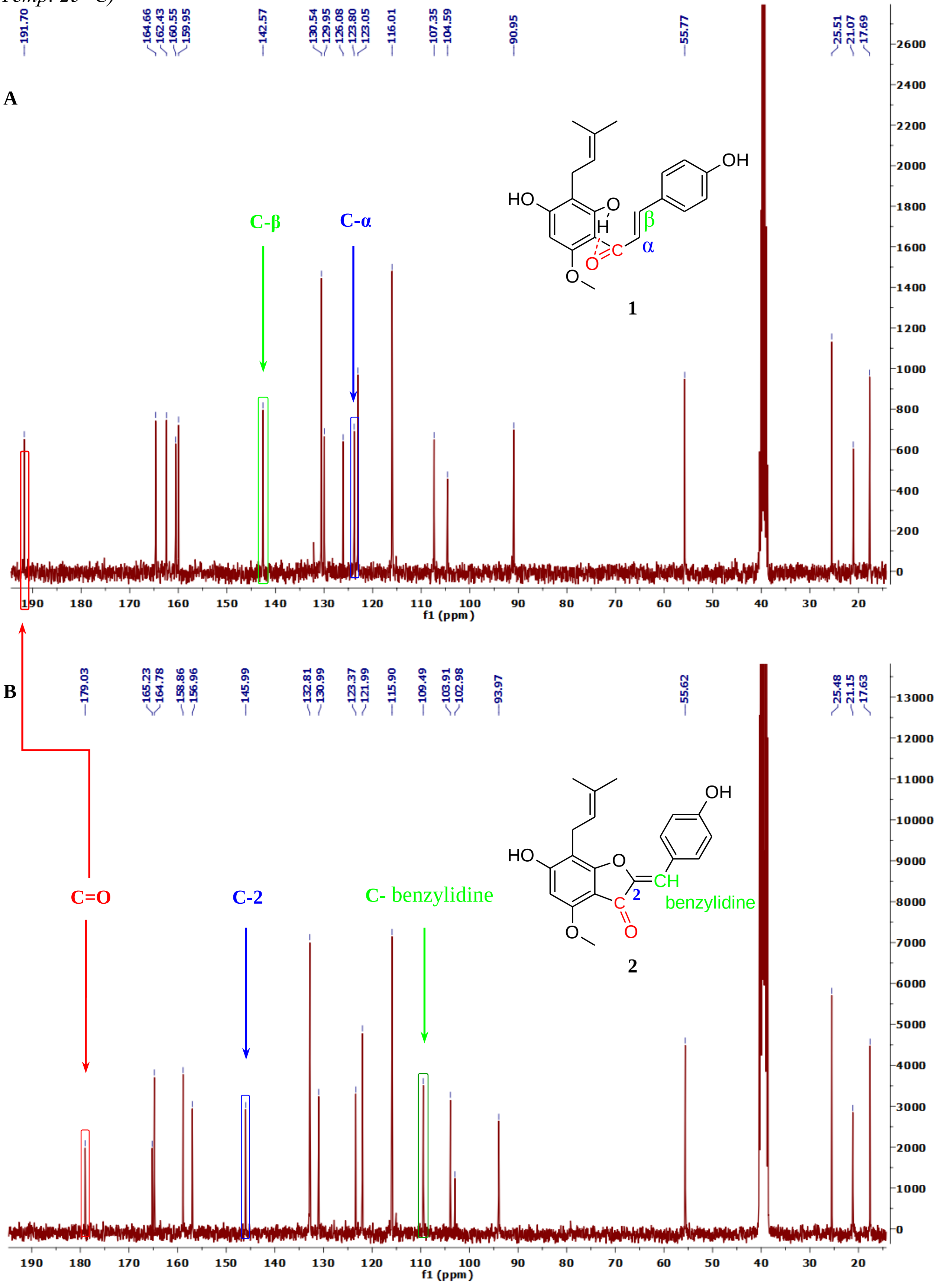

Figure S8. ^{1}H-^{1}H NMR (COSY) spectrum of (Z)-6,4'-dihydroxy-4-methoxy-7-prenylaurone (**3**)(300 MHz/300 MHz, DMSO-d$_6$, Temp. 25°)

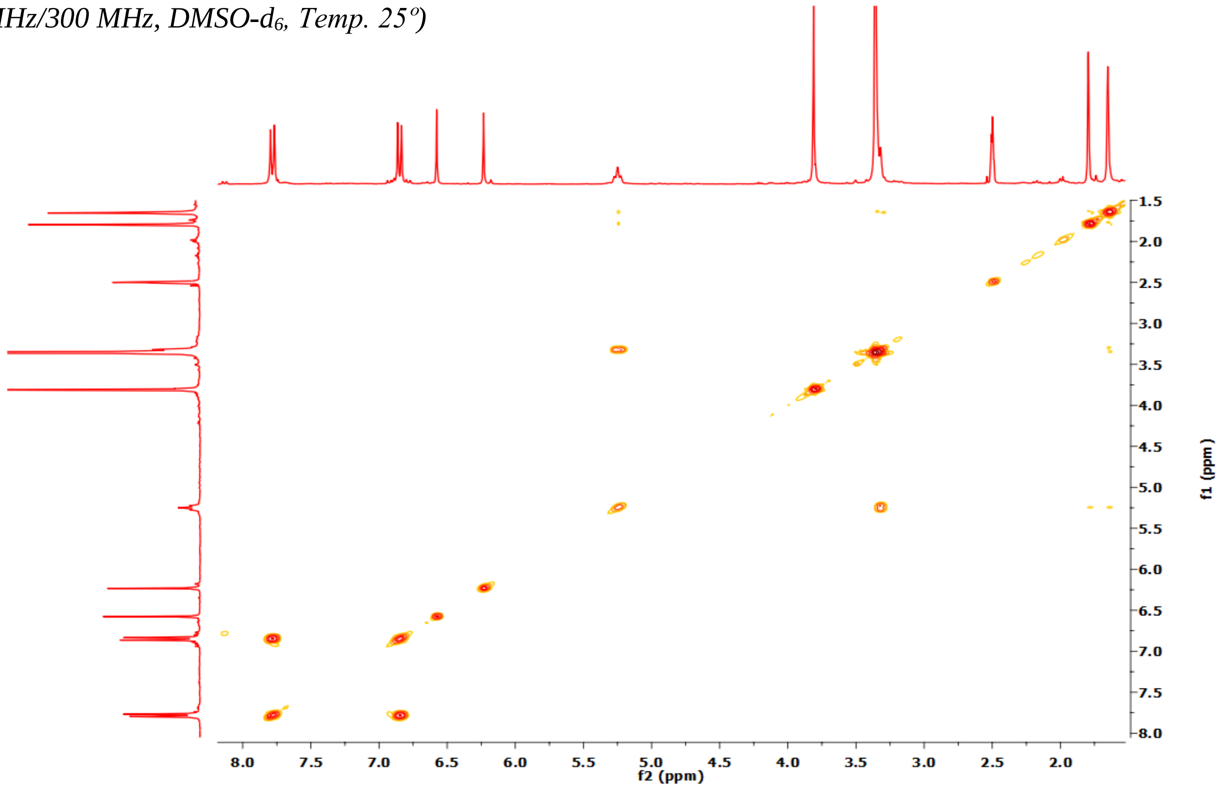

Figure S9. ^{1}H– ^{13}C NMR (HSQC) spectrum of (Z)-6,4'-dihydroxy-4-methoxy-7-prenylaurone (**3**))(300 MHz/75 MHz, DMSO-d$_6$, Temp. 25°)

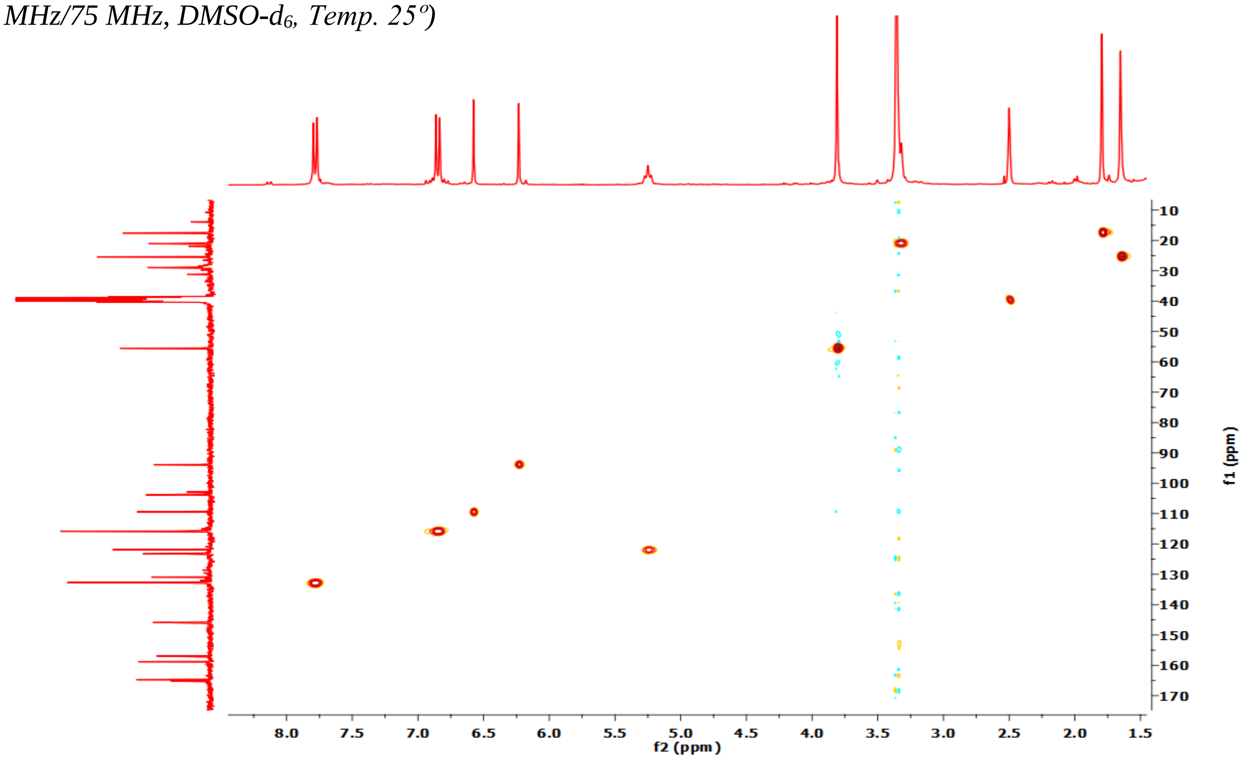

Figure S10. *UV spectrum of α,β-dihydroxanthohumol 4' -O-β-D-glucopyranoside* (**6**) *(MeOH, Temp. 20 °C)*

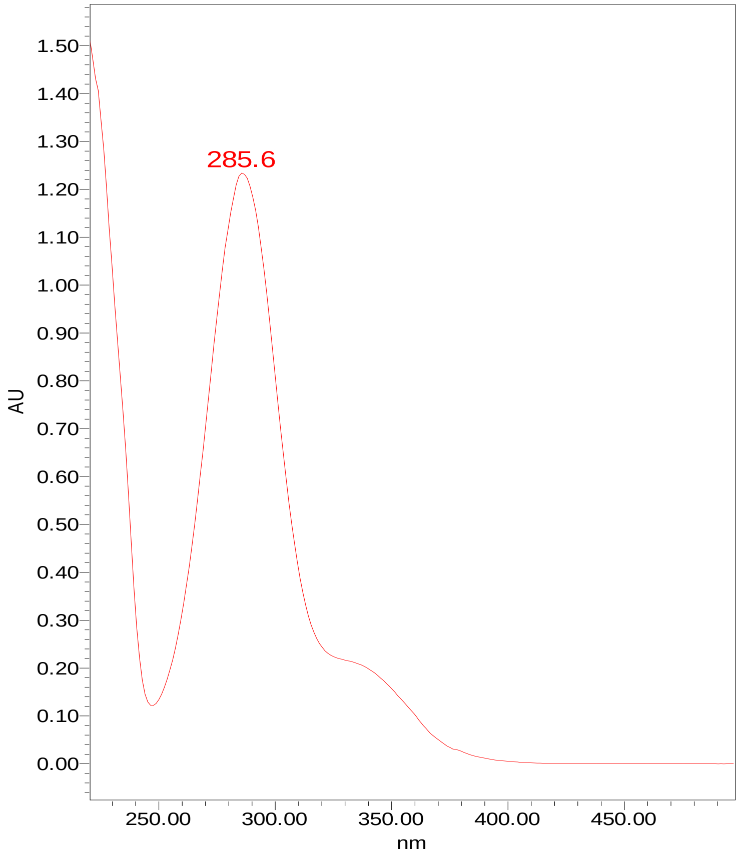

Figure S11. *HRMS spectrum of α,β-dihydroxanthohumol 4' -O-β-D-glucopyranoside* (**6**)

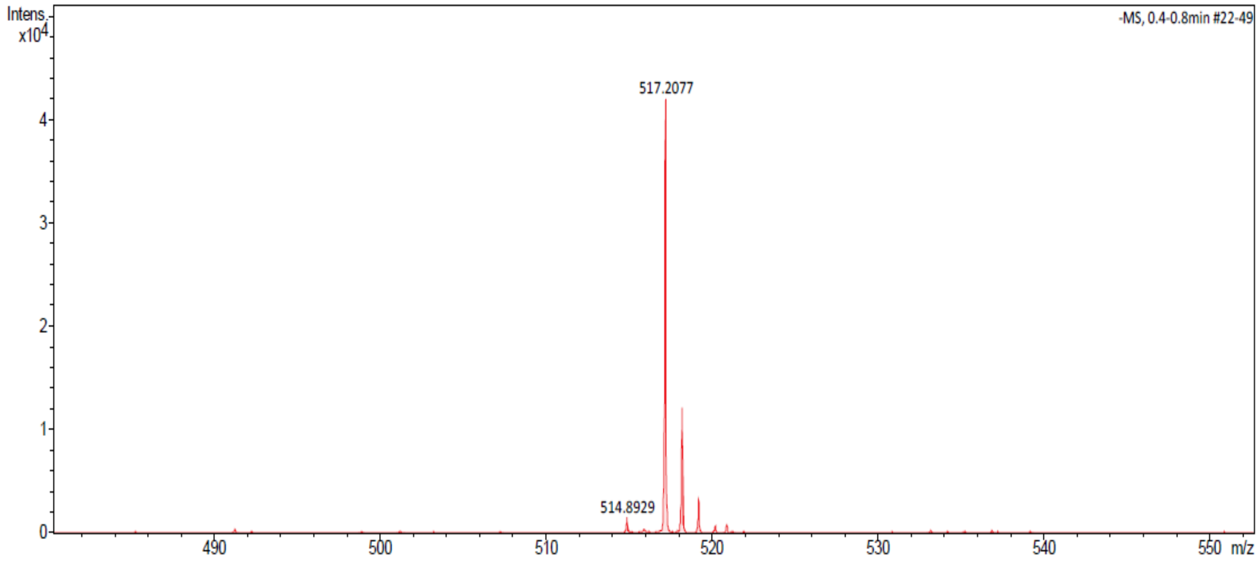

Figure S12. *^{1}H NMR (600 MHz) and ^{13}C NMR (151 MHz) spectra of α,β-dihydroxanthohumol 7-O-β-D-glucopyranoside* ***(6)*** *(Acetone-d$_6$,Temp. 25 ºC)*

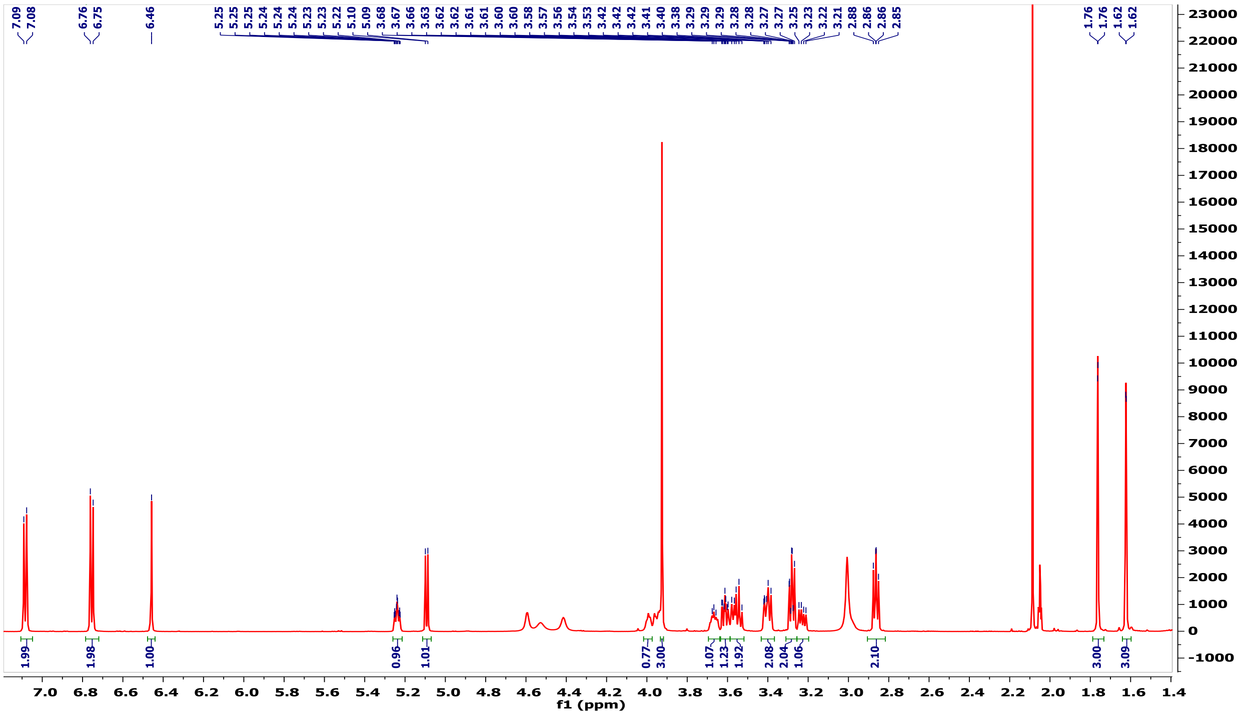

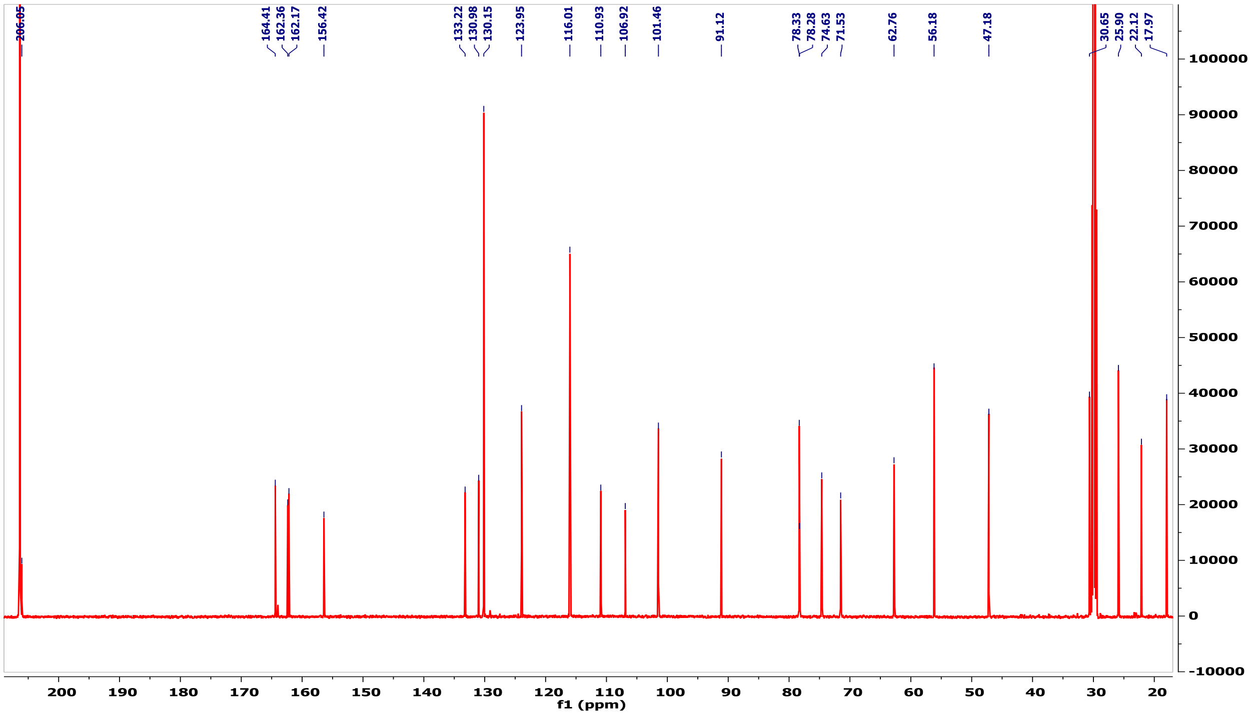

Figure S13. *^{1}H NMR - ^{1}H NMR (COSY) spectrum of α,β-dihydroxanthohumol 7-O-β-D-glucopyranoside* ***(6)*** *)(600 MHz/600 MHz, Acetone-d_6, Temp. 25°)*

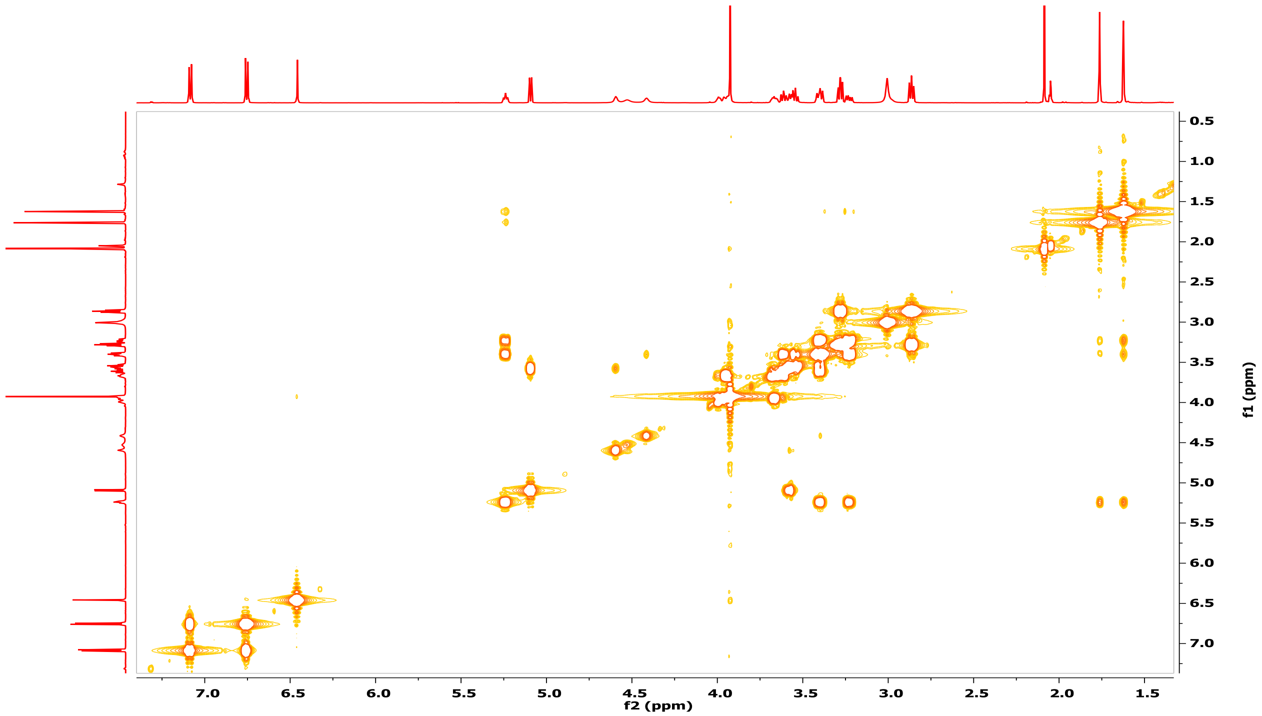

Figure S14. *^{1}H NMR - ^{13}C NMR (HSQC) spectrum of α,β-dihydroxanthohumol 7-O-β-D-glucopyranoside* ***(6)*** *)(600 MHz/151 MHz, Acetone-d_6, Temp. 25°)*

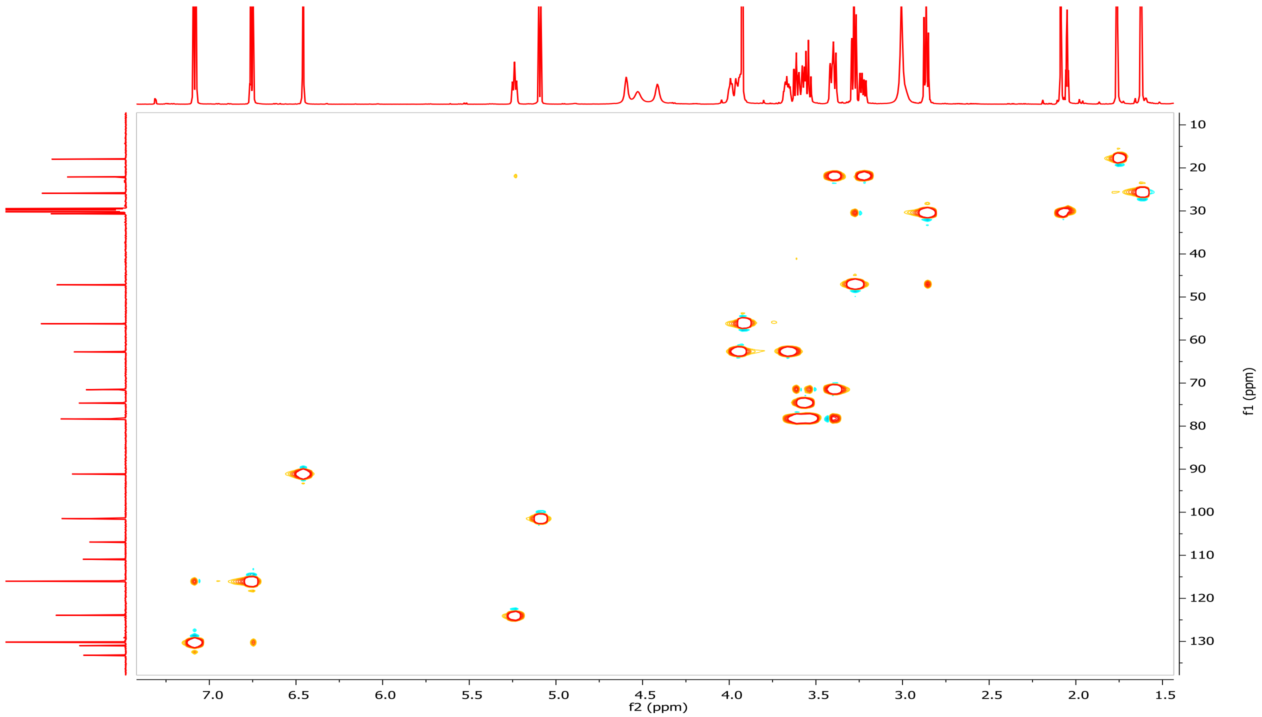

Figure S15. *UV spectrum of α,β-dihydroxanthohumol 7-O-β-D-(4'''-O-methyl)glucopyranoside* ***(7)*** *(MeOH, Temp. 20 °C)*

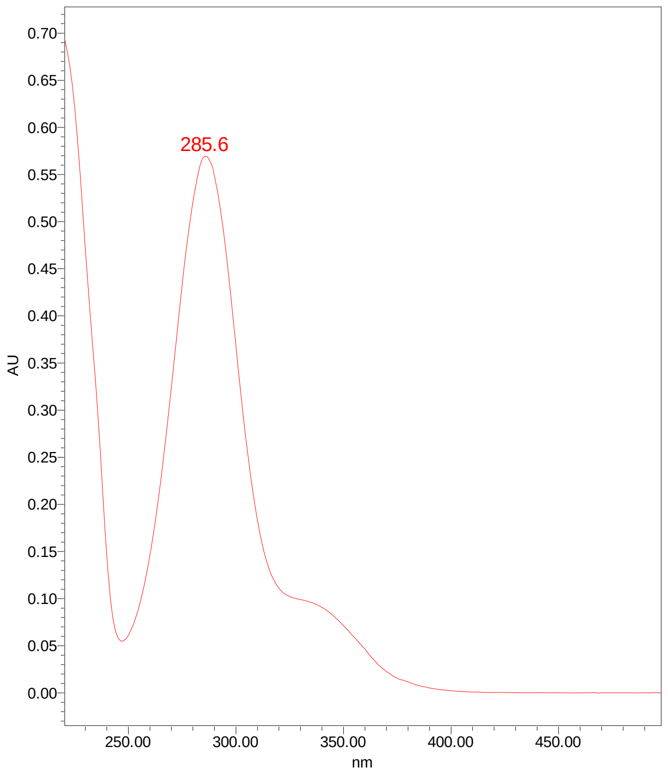

Figure S16. *HRMS spectrum of α,β-dihydroxanthohumol 7-O-β-D-(4'''-O-methyl)glucopyranoside* ***(7)***

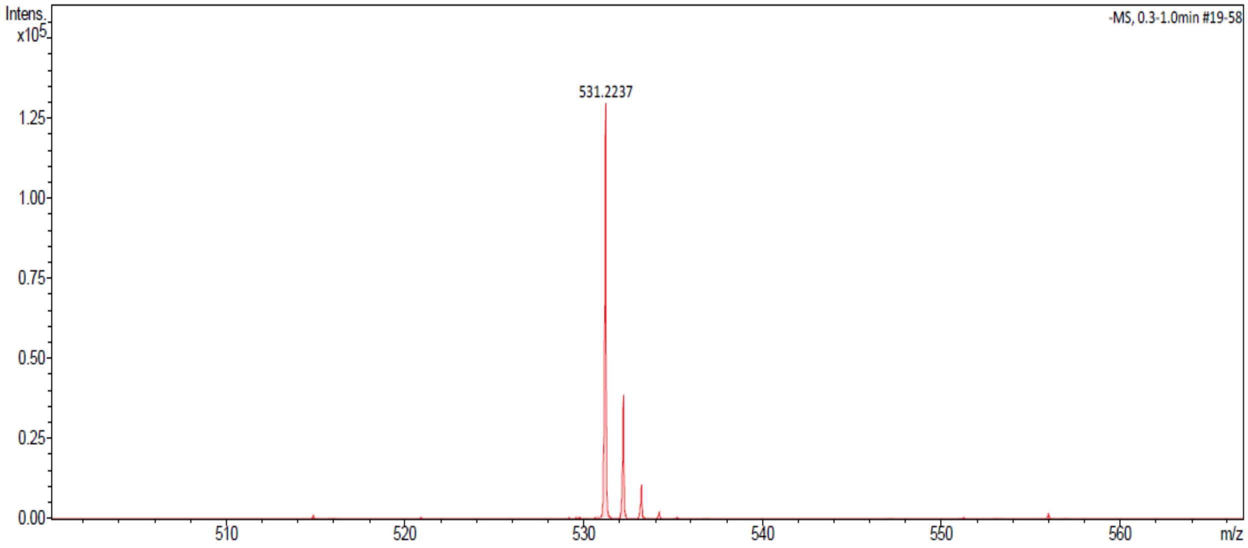

Figure S17. *^{1}H NMR (600 MHz) and ^{13}C NMR (151 MHz) spectra of α,β-dihydroxanthohumol 7-O-β-D-(4'''-O-methyl)glucopyranoside* ***(7)*** *(Acetone-d_6, Temp. 25 ºC).*

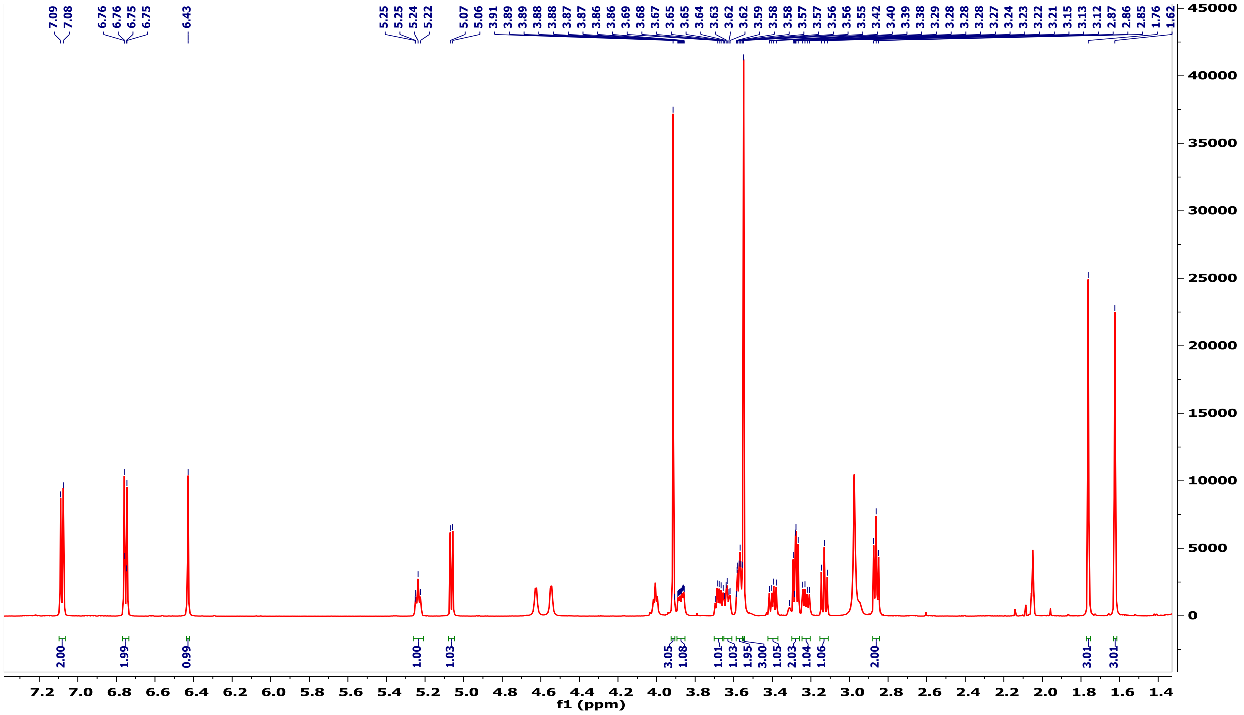

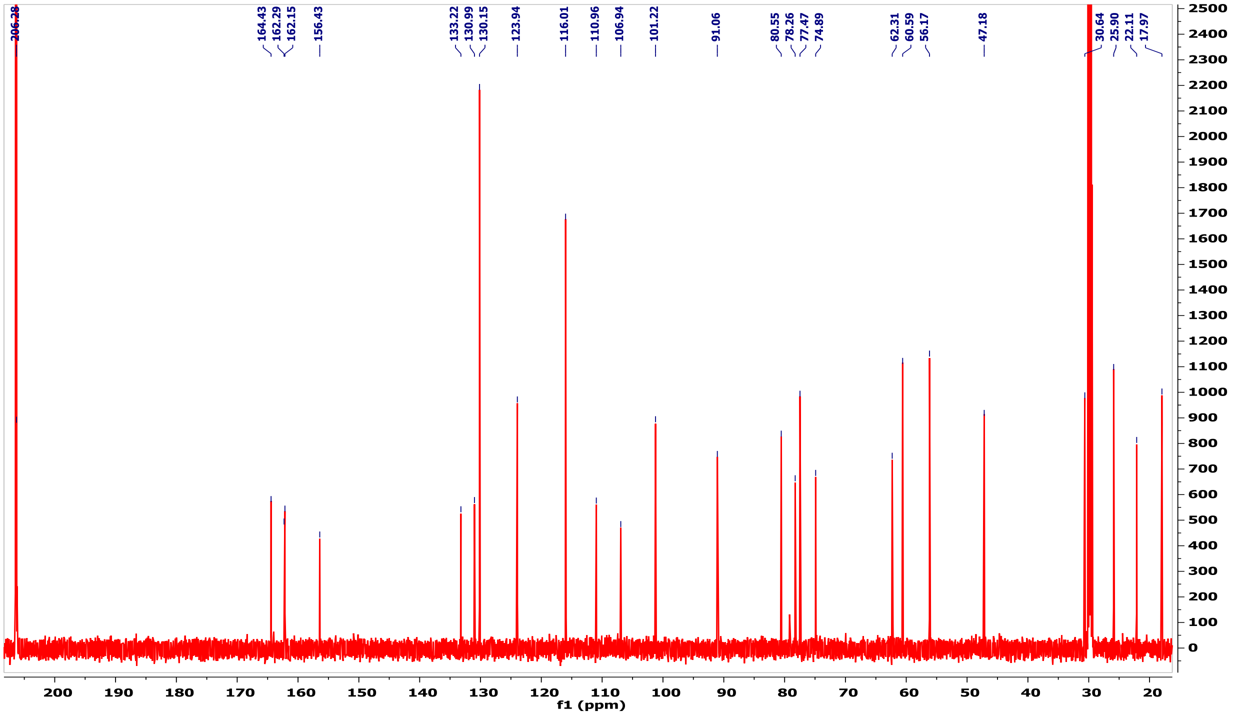

Figure S18. ^{1}*H NMR -* ^{1}*H NMR (COSY) spectrum of α,β-dihydroxanthohumol 7-O-β-D-(4'''-O-methyl)glucopyranoside* ***(7)*** *(600 MHz/600 MHz, Acetone-d$_6$, Temp. 25°)*

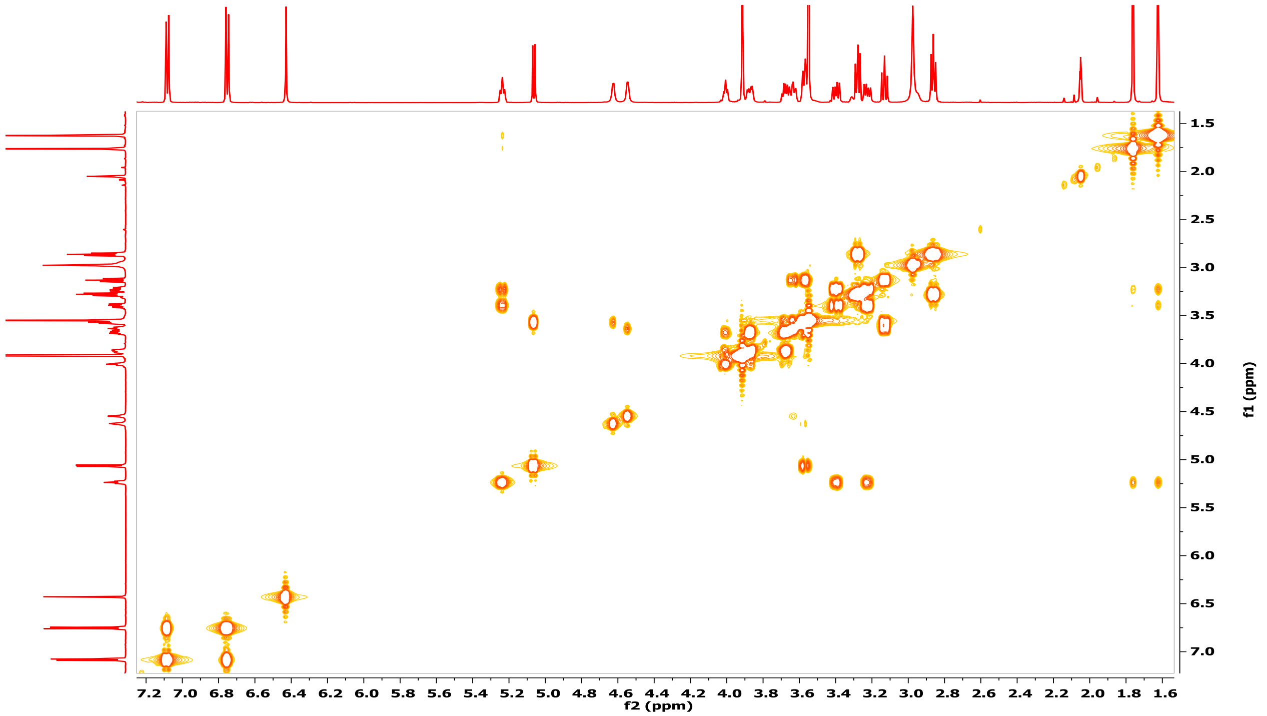

Figure S19. ^{1}*H NMR -* ^{13}*C NMR (HSQC) spectrum of α,β-dihydroxanthohumol 7-O-β-D-(4'''-O-methyl)glucopyranoside* ***(7)*** *(600 MHz/151 MHz, Acetone-d$_6$, Temp. 25°)*

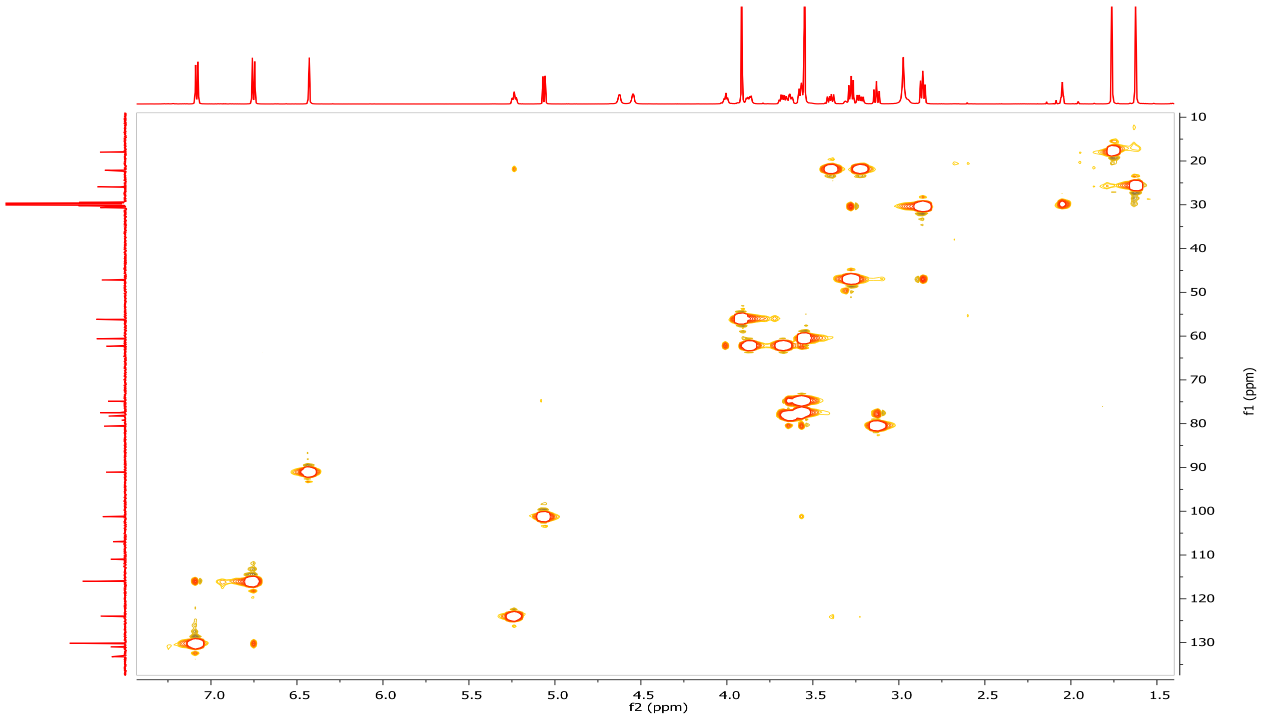

Figure S20. *UV spectrum of (Z)-6,4'-dihydroxy-4-methoxy-7-prenylaurone 6-O-β-D-glucopyranoside* ***(8)*** *(MeOH, Temp. 20 °C)*

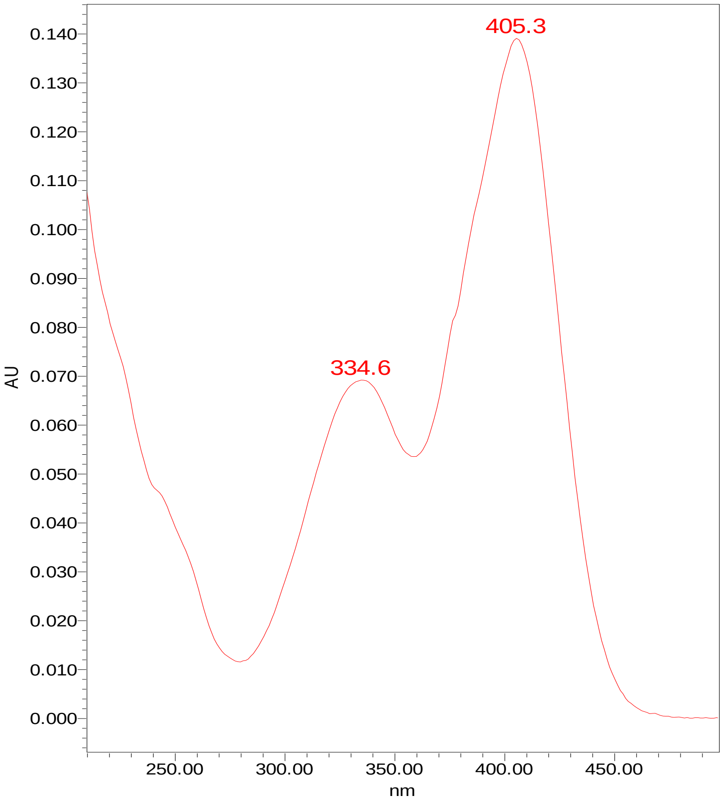

Figure S21. *HRMS spectrum of (Z)-6,4'-dihydroxy-4-methoxy-7-prenylaurone 6-O-β-D-glucopyranoside* ***(8)***

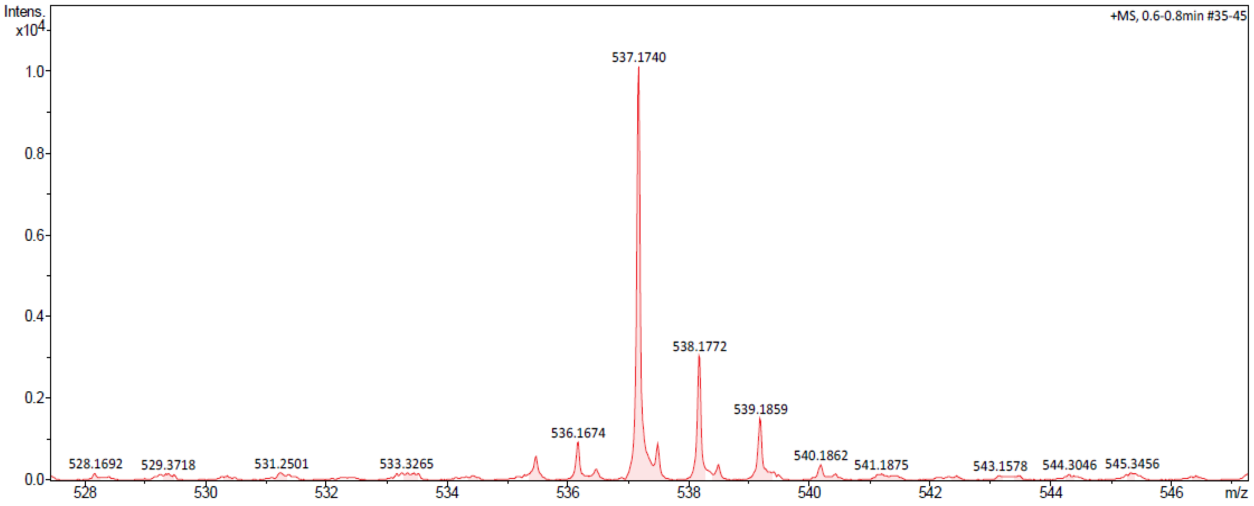

Figure S22. [1]*H NMR (600 MHz) and* [13]*C NMR (151 MHz) spectra of (Z)-6,4'-dihydroxy-4-methoxy-7-prenylaurone 6-O-β-D-glucopyranoside* ***(8)*** *CD_3OD, Temp. 25 ºC)*

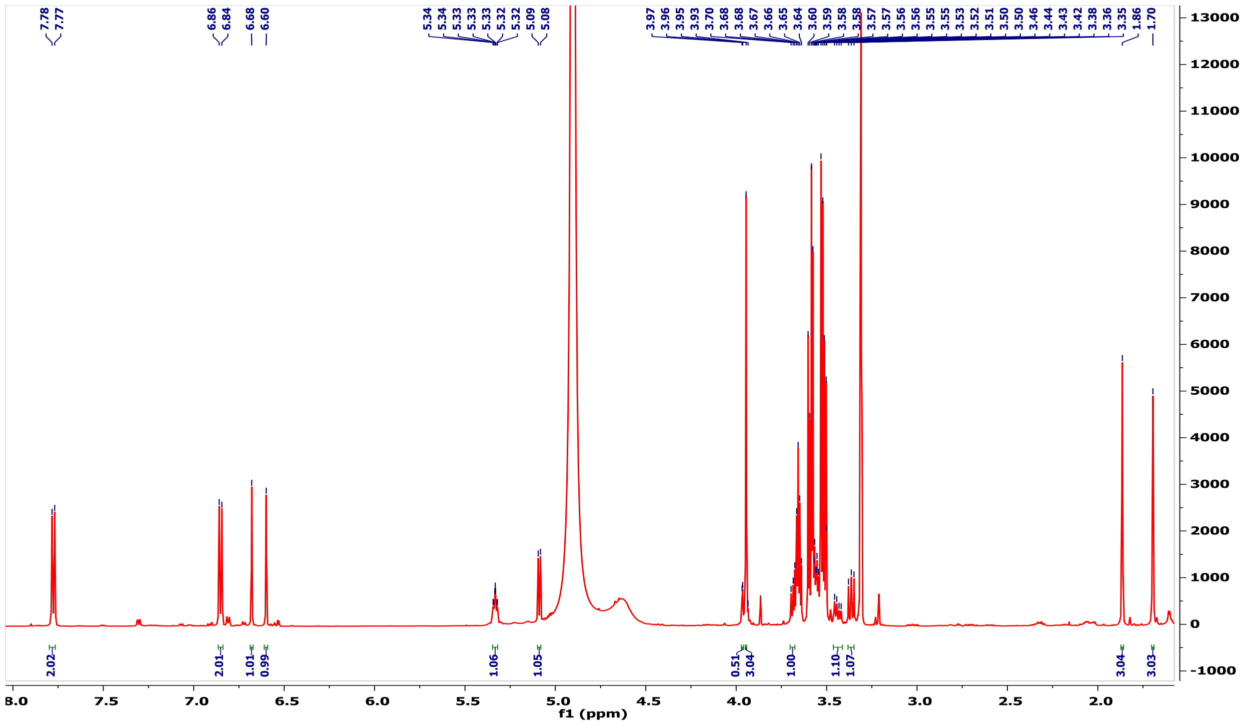

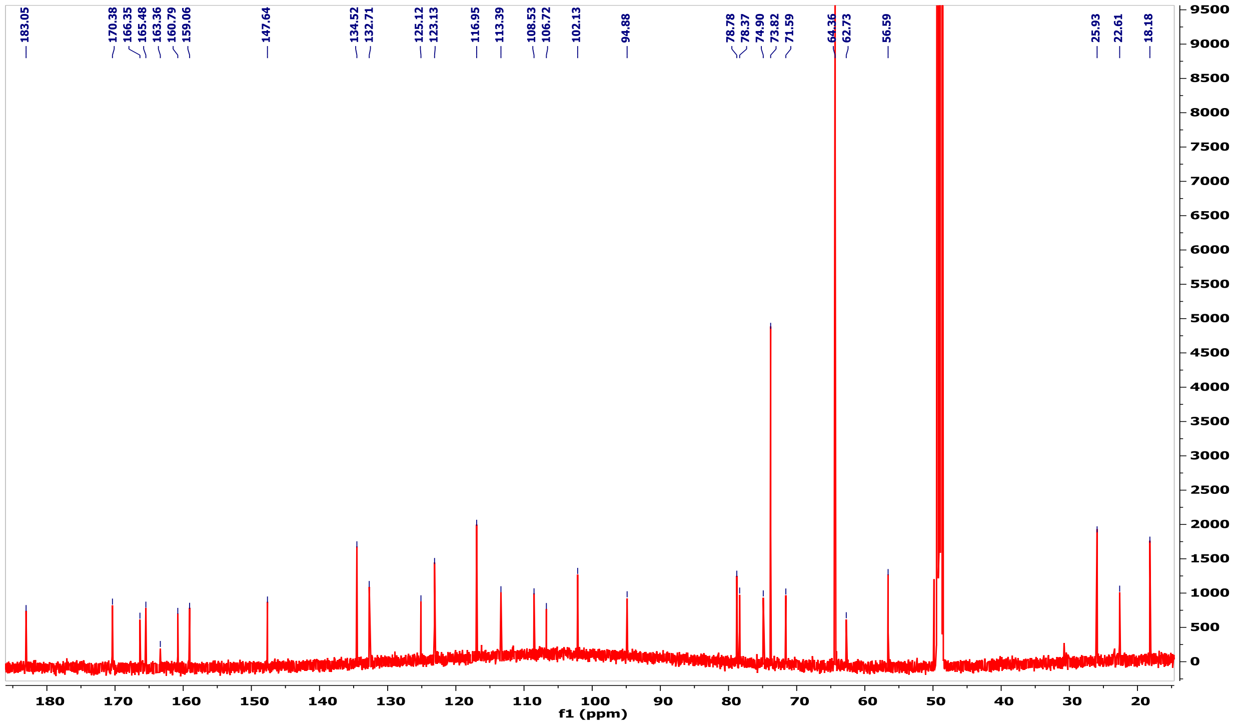

Figure S23. ^{1}*H NMR -* ^{1}*H NMR (COSY) spectrum of (Z)-6,4'-dihydroxy-4-methoxy-7-prenylaurone 6-O-β-D-glucopyranoside* ***(8)*** *(600 MHz/600 MHz, CD_3OD, Temp. 25 ºC)*

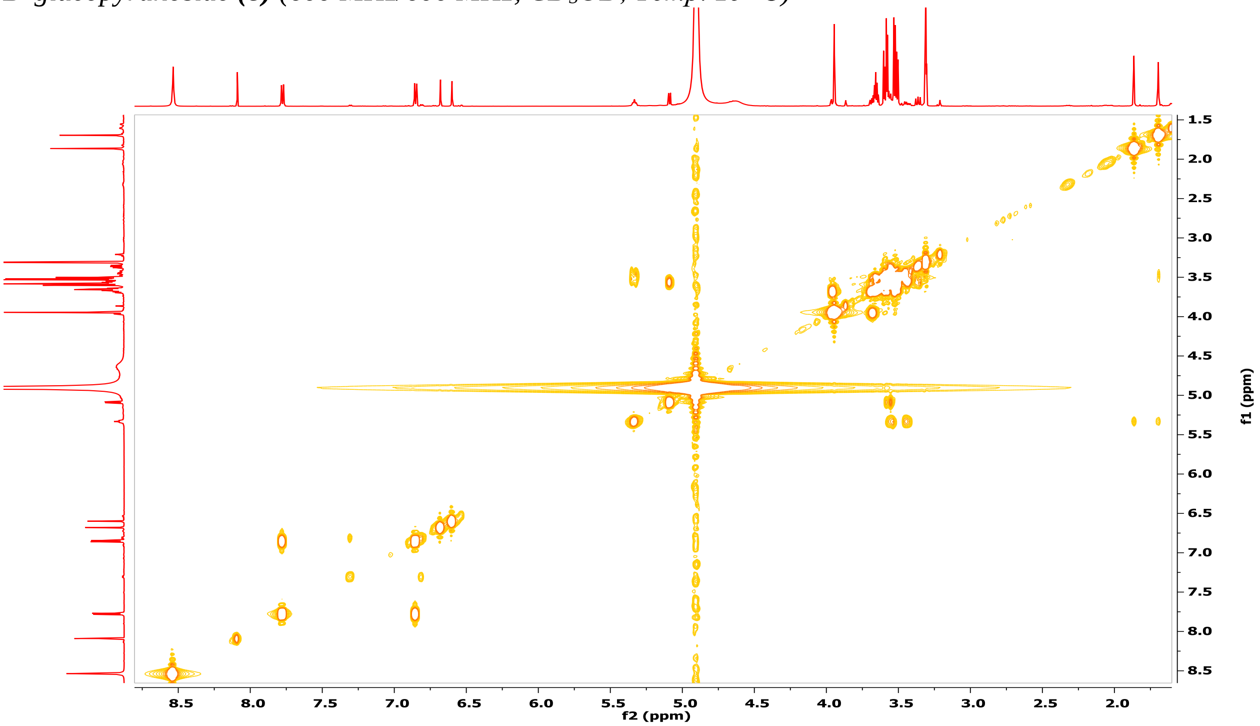

Figure S24. ^{1}*H NMR -* ^{13}*C NMR (HSQC)spectrum of (Z)-6,4'-dihydroxy-4-methoxy-7-prenylaurone 6-O-β-D-glucopyranoside* ***(8)*** *(600 MHz/151 MHz, CD_3OD, Temp. 25 ºC)*

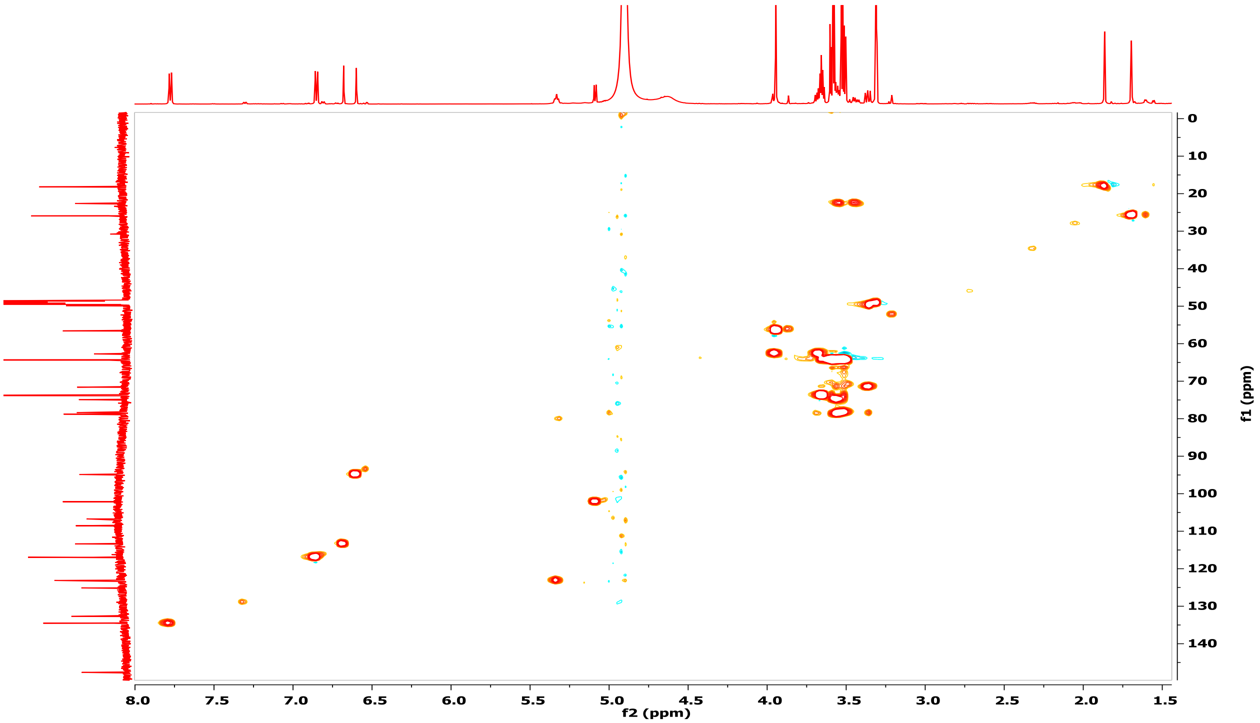

Figure S25. *UV spectrum of (Z)-6,4'-dihydroxy-4-methoxy-7-prenylaurone 6-O-β-D-(4'''-O-methyl)glucopyranoside (**9**)(MeOH, Temp. 20 °C)*

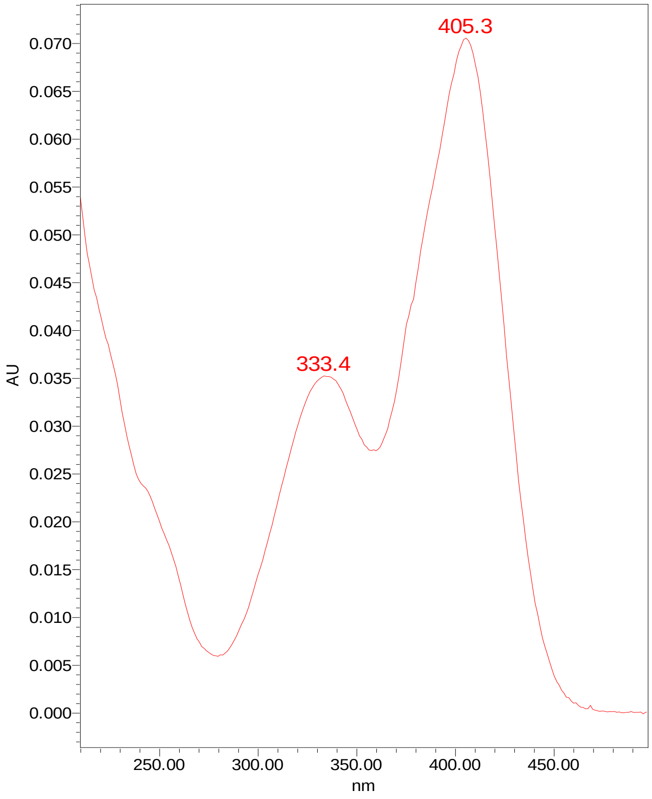

Figure S26. *HRMS spectrum of (Z)-6,4'-dihydroxy-4-methoxy-7-prenylaurone 6-O-β-D-(4'''-O-methyl)glucopyranoside (**9**)*

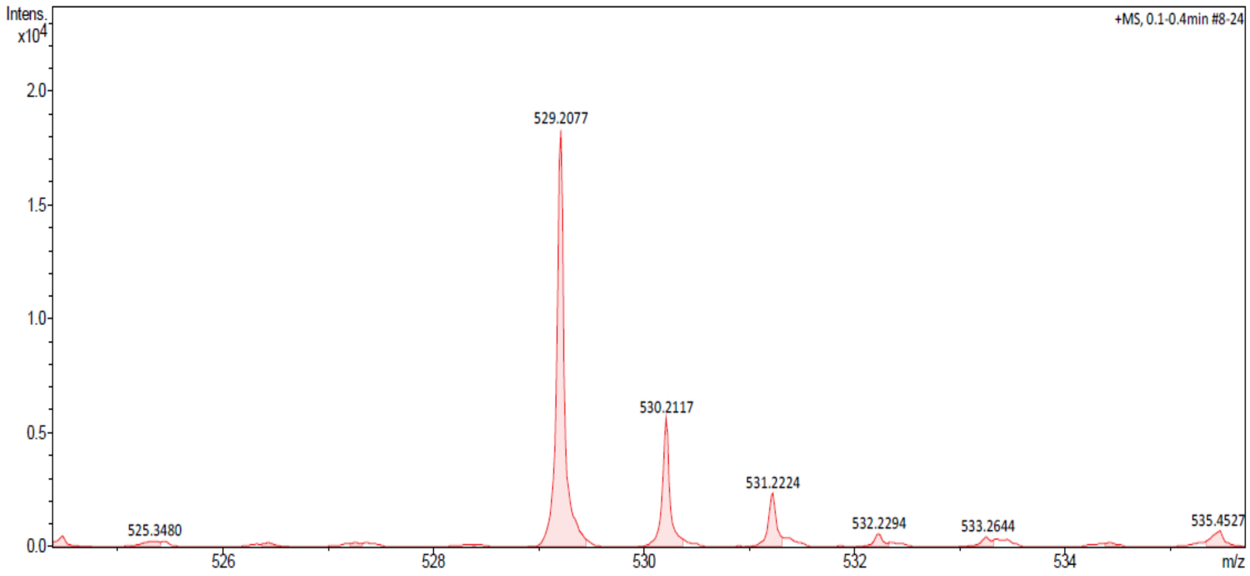

Figure S27. *^{1}H NMR (300 MHz) and ^{13}C NMR (75 MHz,) spectra of (Z)-6,4'-dihydroxy-4-methoxy-7-prenylaurone 6-O-β-D-(4'''-O-methyl)glucopyranoside* (**9**) *(DMSO-d_6, Temp. 25 ºC)*

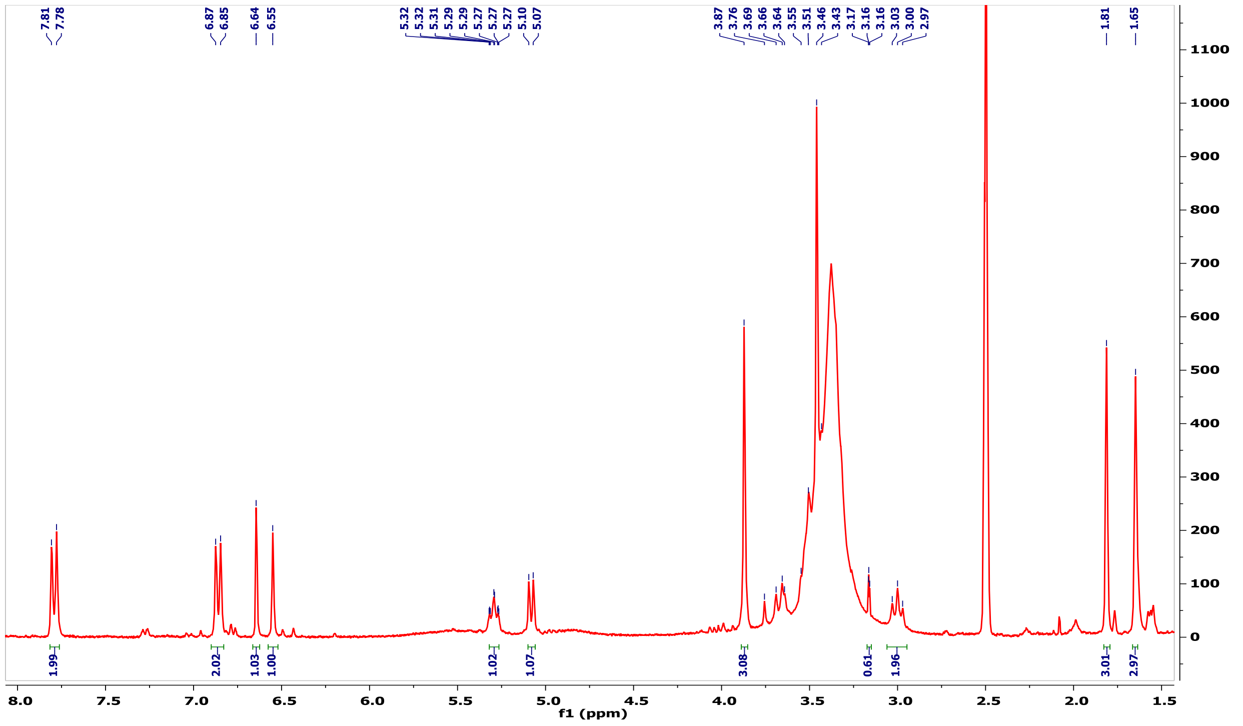

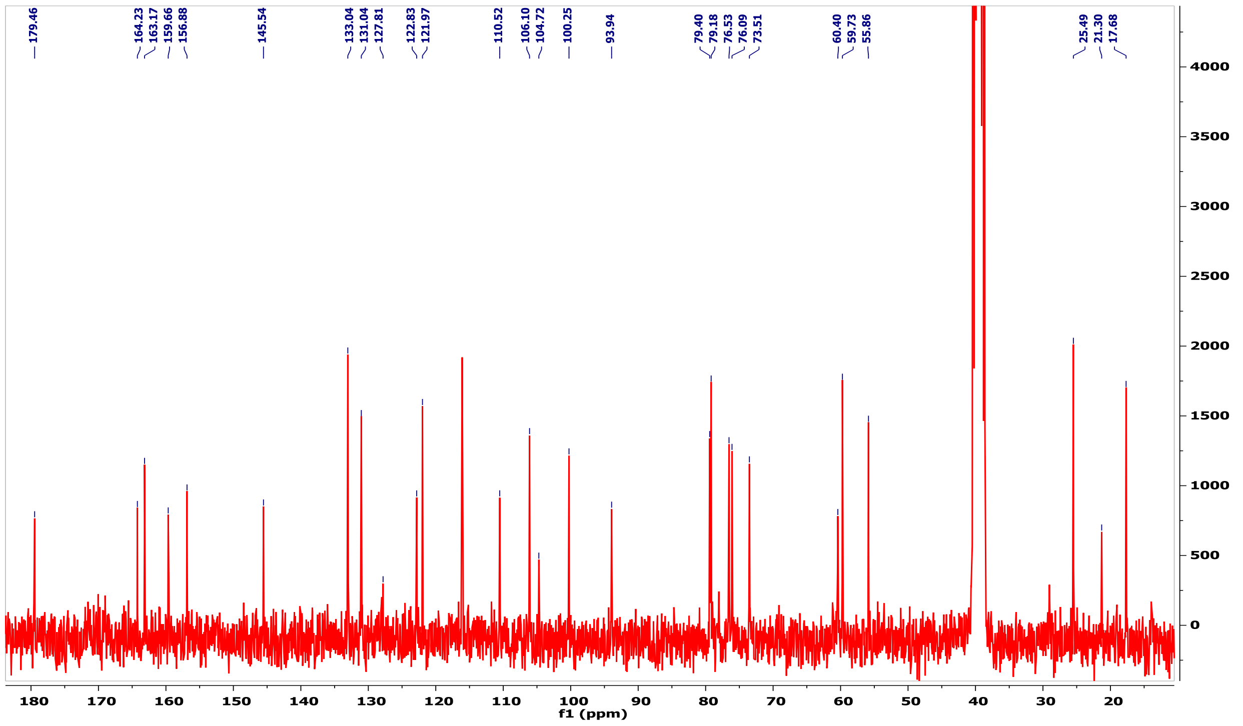

Figure S28. ^{1}*H NMR -* ^{1}*H NMR (COSY) spectrum of (Z)-6,4'-dihydroxy-4-methoxy-7-prenylaurone 6-O-β-D-(4'''-O-methyl)glucopyranoside* **(9)** *) (600 MHz/600 MHz, DMSO-d$_6$, Temp. 25 ºC)*

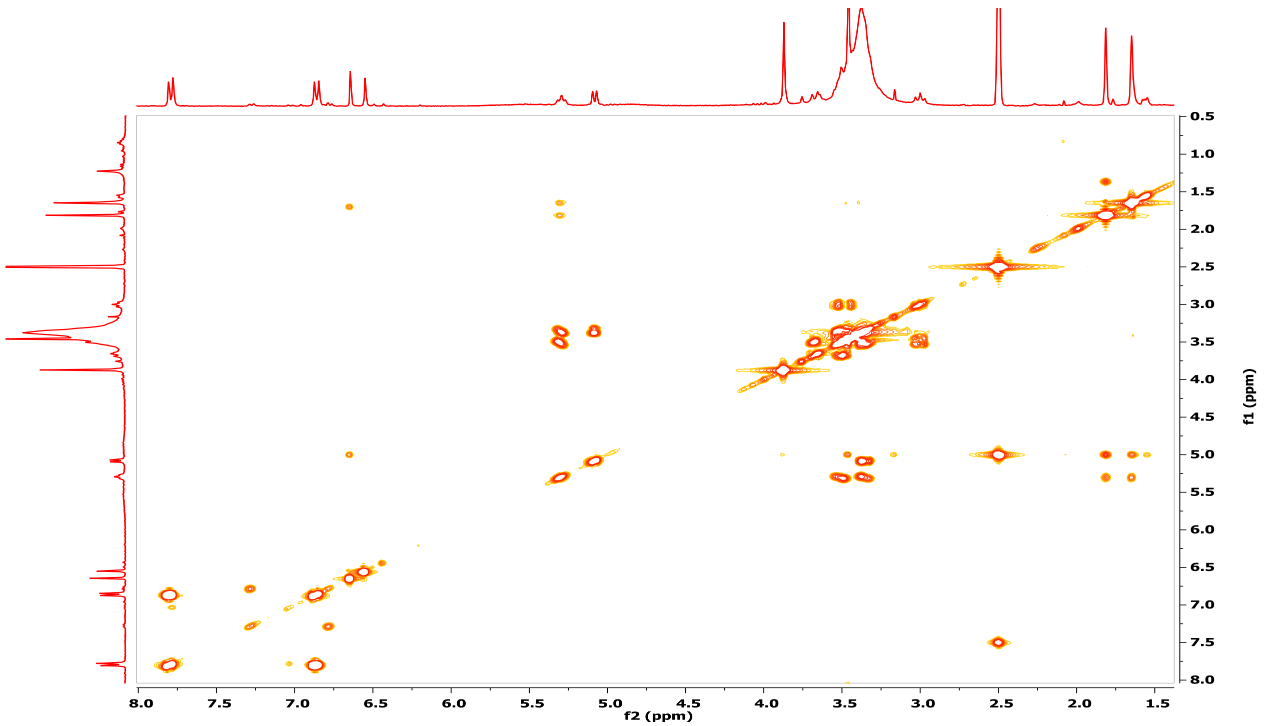

Figure S29. ^{1}*H NMR -* ^{13}*C NMR (HSQC) spectrum of (Z)-6,4'-dihydroxy-4-methoxy-7-prenylaurone 6-O-β-D-(4'''-O-methyl)glucopyranoside* **(9)** *(600 MHz/151 MHz, DMSO-d$_6$, Temp. 25 ºC)*

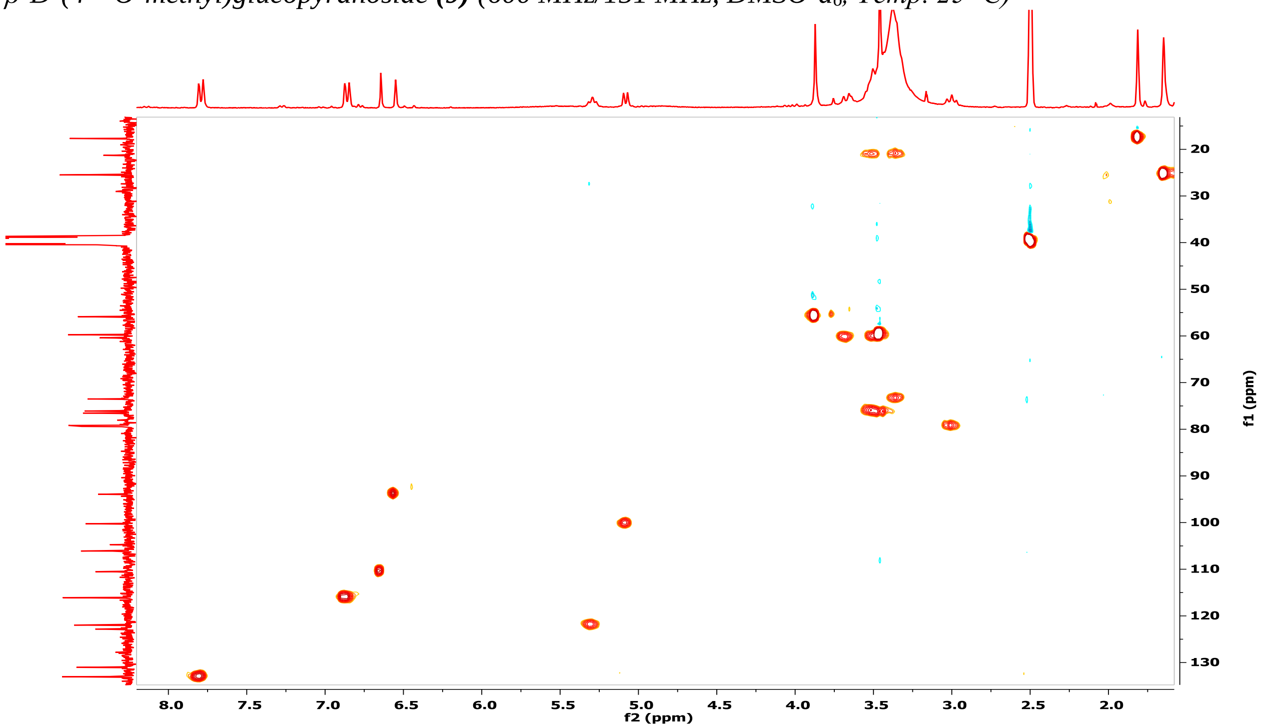

Supplement: Supplementary file 1 [file molecules-22-01230-s001.pdf]
